# Nonintuitive Immunogenicity and Plasticity of Alpha-Synuclein Conformers: A Paradigm for Smart Delivery of Neuro-Immunotherapeutics

**DOI:** 10.3390/pharmaceutics16050609

**Published:** 2024-04-30

**Authors:** Amos Abioye, Damilare Akintade, James Mitchell, Simisade Olorode, Adeboye Adejare

**Affiliations:** 1 College of Pharmacy and Health Sciences, Belmont University, Nashville, TN 37212, USA; 2 Department of Biomedical Sciences, School of Health, Leeds Beckett University, Leeds LS1 3HE, UK; d.akintade@leedsbeckett.ac.uk (D.A.); j.mitchell4828@student.leedsbeckett.ac.uk (J.M.); s.olorode2836@student.leedsbeckett.ac.uk (S.O.); 3 Department of Pharmaceutical Sciences, Philadelphia College of Pharmacy, Saint Joseph’s University, Philadelphia, PA 19131, USA; aadejare@sju.edu

**Keywords:** alpha-synuclein conformers, self-assemblies, neuroinflammation, nonintuitive behavior, immunogenicity, neuroprotection, immunotherapeutics, Parkinson’s disease, nanoparticles, brain drug delivery

## Abstract

Despite the extensive research successes and continuous developments in modern medicine in terms of diagnosis, prevention, and treatment, the lack of clinically useful disease-modifying drugs or immunotherapeutic agents that can successfully treat or prevent neurodegenerative diseases is an ongoing challenge. To date, only one of the 244 drugs in clinical trials for the treatment of neurodegenerative diseases has been approved in the past decade, indicating a failure rate of 99.6%. In corollary, the approved monoclonal antibody did not demonstrate significant cognitive benefits. Thus, the prevalence of neurodegenerative diseases is increasing rapidly. Therefore, there is an urgent need for creative approaches to identifying and testing biomarkers for better diagnosis, prevention, and disease-modifying strategies for the treatment of neurodegenerative diseases. Overexpression of the endogenous α-synuclein has been identified as the driving force for the formation of the pathogenic α-synuclein (α-Syn) conformers, resulting in neuroinflammation, hypersensitivity, endogenous homeostatic responses, oxidative dysfunction, and degeneration of dopaminergic neurons in Parkinson’s disease (PD). However, the conformational plasticity of α-Syn proffers that a certain level of α-Syn is essential for the survival of neurons. Thus, it exerts both neuroprotective and neurotoxic (regulatory) functions on neighboring neuronal cells. Furthermore, the aberrant metastable α-Syn conformers may be subtle and difficult to detect but may trigger cellular and molecular events including immune responses. It is well documented in literature that the misfolded α-Syn and its conformers that are released into the extracellular space from damaged or dead neurons trigger the innate and adaptive immune responses in PD. Thus, in this review, we discuss the nonintuitive plasticity and immunogenicity of the α-Syn conformers in the brain immune cells and their physiological and pathological consequences on the neuroimmune responses including neuroinflammation, homeostatic remodeling, and cell-specific interactions that promote neuroprotection in PD. We also critically reviewed the novel strategies for immunotherapeutic delivery interventions in PD pathogenesis including immunotherapeutic targets and potential nanoparticle-based smart drug delivery systems. It is envisioned that a greater understanding of the nonintuitive immunogenicity of aberrant α-Syn conformers in the brain’s microenvironment would provide a platform for identifying valid therapeutic targets and developing smart brain delivery systems for clinically effective disease-modifying immunotherapeutics that can aid in the prevention and treatment of PD in the future.

## 1. Introduction

Approximately one million Americans are living with Parkinson’s disease (PD), and this number is expected to rise to 1.2 million by 2030 [1]. The direct and indirect costs of PD are estimated to be about 52 billion U.S. dollars every year [2]. Over 10 million people are living with PD worldwide, and it is the most common movement disorder, second in prevalence only to Alzheimer’s disease (AD) among the neurodegenerative diseases [1,3]. PD prevalence has increased steadily over the years from 2.5 million people in 1990 to 6.1 million in 2016 and 10 million in 2022. It is more disturbing to note that a three-fold increase in the prevalence of PD, up to 17.5 million, is anticipated by 2040 [4,5]. In the U.S., the Parkinson’s Foundation reported a steep 50% annual increase in PD prevalence from 60,000 diagnoses in 2021 to 90,000 diagnoses in 2022 [6]. The primary risk factor for PD is age, affecting approximately 5% of the population above 60 years of age; the world’s aging population is expected to double over the next three decades up to 1.6 billion in 2050 because people are living longer and healthier lives in this generation [7]. Other risk factors include environmental challenges and genetic vulnerability. Although significant advances in symptomatic therapy for PD patients have been made since the condition was first diagnosed over 200 years ago, medications targeted to prevent the progression or change the course of the disease (disease-modifying therapeutics) have remained elusive [8,9]. Therefore, there is an urgent need for creative approaches to combating the menace of neurodegenerative diseases including PD.

PD is characterized by a distinct aging-independent gradual but extensive (60–80%) loss of dopaminergic neurons of the substantia nigra pars compacta (SNpc) of the basal ganglia [10], including a progressive loss of structure and function or death of neurons leading to severe depletion of striatal dopamine (dopaminergic neurodegeneration) [11,12]. This loss of dopamine leads to the manifestation of cognitive deficits and various characteristic motor symptoms such as slowed movement (bradykinesia), muscular rigidity or stiffness, resting tremor, and postural and gait abnormality [13]. It may also include the presence of extracellular melanin released from degenerating neurons, reactive gliosis, and eosinophil-laden inclusion bodies (Lewy bodies) in residual substantia nigra neurons [11,14,15]. Furthermore, other populations of neuromelanin-containing neurons in the brainstem and basal forebrain do degenerate and aggregate to form Lewy bodies similar to the neurons in the substantia nigra. These Lewy bodies possibly account for other clinically important psychiatric symptoms of PD like depression, anxiety, hallucination, delusion, apathy and anhedonia, impulsive and compulsive behaviors, cognitive dysfunction, sleep disorders, seborrhoea, autonomic instability, and dementia which can significantly affect the person’s quality of life [3,16].

Although depleted dopamine levels are primarily due to the loss of presynaptic neurons, a compensatory upregulation of dopamine receptors in the postsynaptic neurons has been reported [17]. Therefore, the initial choice of PD therapies includes the administration of levodopa for dopamine replacement and dopamine agonists for the upregulated dopamine receptors [18]. Both of these therapies aim to ameliorate the motor impairment symptoms of PD; however, they do not offer any curative outcomes [19]. PD is also characterized by the buildup of misfolded or mutated α-synuclein (α-Syn) SNCA gene and α-Syn conformers like fibrils in the surviving neurons, which play a significant role in the formation of Lewy bodies and Lewy neurites through intracellular interactions [20]. Underlining these characteristics is the potential immunogenicity of α-Syn conformers and their intracellular aggregates, which may trigger a series of immunological responses in the brain including the production of specific α-Syn conformer antibodies. Thus, specific monoclonal α-Syn antibodies can be developed to facilitate the degradation of α-Syn aggregates [21], prevent cell-to-cell migration [22], and ameliorate behavioral deficits in animal models [23]. There has been significant progress in the identification of mutation motifs in the SNCA gene that encodes α-Syn in familial PD, as demonstrated in a groundbreaking report by Polymeropoulos et al. [24]. There has also been significant progress in understanding the presence of α-Syn as the major protein component of the Lewy body in sporadic PD patients’ postmortem brain [25]. However, presently, the focus on the potential immunogenicity of α-Syn conformers is limited, and there are no neuroprotective or neuroregenerative therapies for the prevention or treatment of these chronic neurodegenerative disorders [24,25]. Hence, the disease is untreatable to date.

Several research efforts have been made in curative therapeutic strategies that target the prevention of neuronal loss, replacement of injured or lost neurons through stem cell therapies, use of growth factors to ameliorate neural injuries, prevention of Lewy bodies formation, elimination of misfolded α-Syn aggregates, and modulation of the brain’s microenvironment [26]. However, little or no significant scientific or clinical success has been reported in modern literature because α-Syn has the potential to undergo a profound conformational switch to form insoluble fibrils through multiple soluble and insoluble cytotoxic heterogeneous intermediate oligomeric conformers, polymorphs, and aggregates many of which have not been fully identified or characterized [27]. It is also not clear which of the conformers, polymorphs, or aggregates are most neurotoxic, and the mechanism of polymorphism and structure-pathogenic relationship of the conformer remains elusive. Several studies have suggested that the soluble intermediate conformers are the most cytotoxic species, rather than the insoluble fibrils and their aggregates [28,29]. These soluble intermediate conformers could serve as early biomarkers preceding the neuronal decline, which are not available presently; however, they are transient, dynamic, and very difficult to detect and characterize [27]. Thus, current research efforts to reduce the propagation of PD pathogenesis have been focused on reducing the intracellular burden of α-Syn overexpression and the consequent toxicity of its conformers by targeting the misfolded or aggregated α-Syn through the reduction of α-Syn expression (antisense therapeutics) [30], prevention of toxic α-Syn aggregate formation (anti-aggregation agents) [31,32], use of aggregate sequestering agents [33], use of cellular clearance of toxic forms of α-Syn (autophagy [34] and lysosomal [35] mechanisms), modulation of neuroinflammation processes [36], and elimination of intracellular and extracellular α-Syn aggregates through active [37] and passive [38] immunotherapy. Although many of these strategies have demonstrated potential promise in the preclinical and/or early phase of clinical trials, there are widespread limitations that hinder their large-scale implementation, including poor blood–brain barrier penetration, the potential for off-target effects, and the need to protect the functional role of α-Syn rather than eliminating it completely [29]. Irrespective of the stated approaches in current research, the prominent limitation is the insufficient understanding of the α-Syn structure, dynamics, physiological functions, and the nonintuitive immunogenic behavior of its conformers in PD pathogenesis.

Therefore, the purpose of this review is to explore a deeper understanding of the nonintuitive behavior of α-Syn in which it transitions from a physiologically functional state to pathological conformations and how the immunogenicity of the α-Syn conformers may translate into potential immunotherapeutic agents that can help in early detection, prevention and successful treatment of PD. We have critically reviewed the potential nonintuitive systemic toxigenic effects of α-synuclein conformers, including conformational plasticity, aggregation propensity, phenotypic switching, and immunogenicity-induced inflammatory and immunological responses. This is followed by novel strategies for immunotherapeutic delivery interventions that can address the present limitations in immunotherapeutic approaches. It is envisioned that addressing these knowledge gaps will illuminate our knowledge on why and when the α-Syn functional-pathologic transition occurs, the critical role of α-Syn conformers in neuroprotection and neurotoxicity, their impact on PD pathogenesis, and why some people develop PD while others do not. These findings will provide a robust platform for identifying effective immunotherapeutic targets for the diagnosis, prevention, disease progression monitoring, and assessment of prophylactic and therapeutic strategies for PD.

### Structure–Function Relationship (SFR) of Alpha-Synuclein

Although the exact functional role of α-Syn in the nucleus is not fully understood, it has been proposed to play a role in regulating gene transcription and DNA repair [39] and neuronal differentiation [40]. In corollary, there have been reports of increasing incidents of double-stranded damage to the brain tissues of mice lacking α-Syn [41]. However, the effects of α-Syn conformers in the nucleus and the consequent immune responses in the brain are yet to be evaluated. α-Syn has also been associated with synaptic vesicle transport [42] in neurons, including regulation of neurotransmitters’ trafficking from the synaptic vesicles in healthy neurons, control of vesicle fusion [43], neurotransmitter release, and synaptic plasticity by acting as a molecular chaperone in the formation of α-Syn-SNARE complexes [44]. Synaptic vesicle transport in neurons has been identified in immune responses such as the release of granules (granulocytes), receptor recycling and phagocytosis [45], and the relay of signals between neurons, which are critical for normal brain functions [40]. These α-Syn-SNARE complexes are reported to be critical to the presynaptic release of neurotransmitters including dopamine [42]. It follows that α-Syn may influence the release of dopamine, which controls voluntary and involuntary movements as well as memory and cognitive functions as demonstrated in vivo with SNCA knockout mice [46]. The monomeric α-Syn has been reported to exhibit in vitro inhibition of the aggregation of aggregation-prone proteins like aldolase and glutathione S-transferase [47] and heat-induced aggregation of α-Syn [48], suggesting its regulatory and neuroprotective functional roles. It is also involved in transcriptional regulation, modulation of immune cell maturation and response, and acts as an antimicrobial peptide. However, the molecular mechanisms of these roles are not well understood [40].

**Figure 1:**
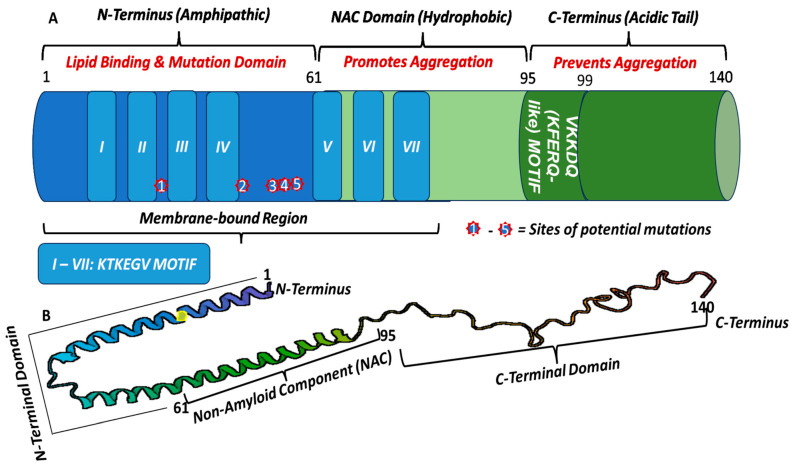
Schematic representation of the functional structure of α-synuclein. (**A**) is the hypothetical representation of α-synuclein indicating its three distinct regions. The blue region represents the N-terminal amphipathic domain containing the amino acid residues affected by the specific α-synuclein gene mutations (1-A30P, 2-E46K, 3-H50Q, 4-G51D, 5-A53E or A53T) commonly associated with Parkinson’s disease. The characteristic primary sequence of imperfect hexameric repeat motif (KTKEGV) in α-synuclein, annotated as I–VII, is responsible for the formation of α-helical structure. The army green region is the non-amyloid component (NAC) domain which promotes α-synuclein aggregation, and the green region is the acidic C-terminus which is the main site for phosphorylation at Ser129. (**B**) is the ribbon structure of the α-synuclein monomer. Created with BioRender.com (accessed on 30 October 2023).

Thus, profiling the molecular structure of alpha-synuclein (α-Syn) would be necessary to understand the link between its nonintuitive behavior and PD pathology, disease progression, and heterogeneity of PD symptoms. Alpha-Synuclein belongs to the family of four soluble proteins, including Alpha-Synuclein (α-Syn), Beta-Synuclein (β-Syn), Gamma-Synuclein (γ-Syn), and Synoretin [49]. Unlike the other members of the Synuclein proteins, α-Syn is abundant in the brain, predominantly at the axon terminals of presynaptic neurons within the frontal cortex, hippocampus, striatum, and olfactory bulb. It is a highly charged presynaptic protein with a molecular weight of 15 kDa consisting of 140 amino acid residues [50]. The polypeptide chain has a complex multifunctional molecular structure with three distinct sequential domains, based on charge and functionality, [51] (Figure 1) that confer conformational flexibility with a propensity to aggregate into several conformers of varying morphology. Thus, understanding the complex structure, sequence, and molecular characteristics of α-Syn could unravel the molecular and structural basis of its physiological functions, neurotoxicity, immunogenicity, degradative pathways, aggregation, and nonintuitive behavior, which are crucial to the development of effective immunotherapeutic strategies for PD.

First, the N-terminal domain (residues 1–60) is a lysine-rich amino terminus which is critical for controlling the α-Syn-membrane interactions (membrane binding) and α-helix formation [49]. It contains seven highly conserved 11 amino acid repeat sequences of lipid-binding apolipoprotein hexamer motifs with KTKEGV consensus [52] that is responsible for the formation of amphiphilic alpha-helical structure, which enables its binding to negatively-charged lipid membranes, including interaction with its carboxy-terminal tail forming self-assemblies and supramolecular complexes [43,53]. Bendor et al. and Lazzaro et al. have reported that this domain facilitates the main mutations (A30P, A53T, E46K, G51D, and H50Q) that are associated with familial PD [43,53]. The authors demonstrated that mutations in this domain weaken the binding of α-Syn to phospholipids, resulting in the formation of oligomeric intermediates with the potential of forming inclusion bodies because amino acid substitution prevents the protein from folding into the regular helical structure. Thus, mutation of the α-Syn has been associated with its misfolding [54]. For example, A30P is a missense mutation where proline (Pro) replaces the 30th amino acid, alanine (Ala), at the N-terminus and exhibits significantly reduced vesicular binding activity, while A53T is a point mutation where the 53rd amino acid, alanine (Ala), is replaced by threonine (Thr) generating α-Syn enriched with β-sheet structure which is prone to fibrillization and aggregation [54]. Similarly, lysine (Lys) replaced glutamic acid (Glu) in E46K, resulting in higher rates of fibrillization because Glu residue prevents β-sheets formation [54]. In general, α-Syn missense mutations in this domain stimulate the conversion of its α-helical structure into β-sheet structures, an index of fibrillization and aggregation, which are the hallmarks of familial PD. Recently, the critical motifs for aggregation were identified as residues 36–42 and 45–47 within the N-terminal domain; thus, deletion of either or both of these motifs prevents α-Syn aggregation and toxicity [55]. In corollary, McGlinchey et al. (2021) developed some N-terminal constructs that inhibit fibril formation, including Gly14-Ala140, Gly36-Ala140, and Gly41-Ala140 [56]. Overall, the amino group in the lysine residue and the N-terminus peptides are highly nucleophilic with high relative abundance. Thus, lysine residue and N-terminus peptides provide a potentially robust template for precision-driven rational design of disease-modifying immunotherapeutic agents for the treatment of PD, including protein-drug bioconjugates, antibody-drug conjugates, amino acid-drug nanoplexes, conjugate vaccines, and smart drug delivery systems.

The second domain is the central hydrophobic region (residues 61–95), also called the non-amyloid component (NAC), which has a strong tendency to form protein assemblies (aggregation) that are rich in β-sheets [57]. β-Syn is a structurally similar isoform of α-Syn with 61% identical amino acid sequence and similar domain organization [58]. However, it does not have central hydrophobic residues 73–83 which form the core of α-Syn aggregates and are responsible for the formation of α-Syn self-aggregates that coalesce to amyloid fibrils and pathological inclusions in PD pathogenesis [57], suggesting a greater stability of the native unfolded β-Syn protein and less propensity to aggregate [20,59]. Nonetheless, β-Syn has been reported to exhibit in vitro neurotoxicity in cultured neuronal cells and progressive in vivo neurodegeneration [60]. Furthermore, immunochemistry studies have shown that α-Syn but not β-Syn is present in the Lewy bodies from the brains of PD patients [25,61]. The NAC forms the dense core of the α-Syn structure with a halo periphery representing the regular folding of α-Syn into a globular structure at a low energy state. Thus, the absence of the NAC domain could lead to misfolding and aggregation [50,62]. Therefore, it suffices to state that the NAC domain is a great target for drug design to control, prevent, and correct misfolding and incidental inclusion of α-Syn as a potential strategy for disease-modifying immunotherapy for PD. On the other hand, the hydrophobic interaction in this domain may lead to oligomerization and aggregation of α-Syn with a string of amino acids at the core of the aggregates, leading to the formation of β-sheet structures and fibrils [50]. Phosphorylation of Ser 87 in this domain also leads to conformational change of α-Syn from α-helix structure to fibrils [50].

Lastly, the C-terminal domain is the highly flexible negatively charged (polar), disordered carboxy-terminal tail which is rich in proline residues and provides resilience that modulates the interaction of α-Syn with other proteins, ligands, metal ions, and chaperones [50]. It contains a calcium binding site and regulates the α-Syn nuclear localization. It also maintains the soluble form of α-Syn by disrupting the potential interactions between the N-terminus and NAC region, which prevents α-Syn aggregation. It suffices to state that the soluble form of α-Syn is relatively nontoxic. However, when it precipitates out of solution, it is vulnerable to misfolding and aggregation, leading to neurotoxicity. Thus, deletion of the C-terminal domain may facilitate α-Syn aggregation and fibril formation.

## 2. Conformational Plasticity and Posttranslational Stability of α-Synuclein Conformers in PD Pathogenesis

The amphipathic nature of α-Syn structural domains confers remarkable conformational plasticity on α-Syn due to long-range intramolecular interaction between the C-terminal tail of α-Syn and the NAC [63] and because the N-terminal regions form heterogeneous, moderately compact (supramolecular stacks), and highly dynamic α-Syn assemblies comprising several conformers of varying sizes [64]. However, the conformational flexibility of α-Syn is site-specific [63,64]. The stabilizing effect of the hydrophobic interaction between the negatively-charged C-terminal and NAC domain and the electrostatic interaction with the N-terminal provide a shielding effect (steric hindrance) and electrostatic repulsion between fibrils of α-Syn. These effects inhibit the process of oligomerization, the formation of the β-sheet structure in the NAC domain, and the consequent α-Syn toxicity [63,64]. This self-autoinhibition of α-Syn aggregation under native conditions constitutes a nonintuitive behavior that is critical to target identification and rational design of immunotherapeutic strategies for the prevention and treatment of PD [65,66,67,68,69]. In corollary, aggregation [70] inducing agents like polycations, metal ions, and pathogenic mutants (A53T or A30P) have been shown to facilitate interaction between the C-terminal and NAC region, reinforcing the significance of these interactions in modulating the propensity of α-Syn-induced aggregation and its consequences [64].

To understand the structural stability and structure–function–toxicity relationship of α-Syn conformers, several techniques have been utilized to investigate the extensive α-Syn posttranslational modifications (PTMs) that contribute to PD pathogenesis [71,72], including glycation, ubiquitination, oxidation, nitrosylation, acetylation, glycosylation and more importantly phosphorylation [70,73,74]. Lee et al. (2011) asserted that increased α-synuclein phosphatase activity was protective against α-Syn-mediated neurotoxicity, suggesting a causal detrimental role of α-Syn in the pathogenesis of PD [75]. Similarly, phosphorylation of α-Syn at serine residue 129 (S129) indicates its significant role in LB formation, increasing the formation of soluble α-Syn oligomer, which leads to neurotoxicity, whereas phosphorylation of α-Syn at the tyrosine residue 125 (Y125) exhibits neuroprotection in Drosophila model [76,77]. In similar studies, Zhou et al. have demonstrated that oxidation of methionine disrupts fibrils formation and enhances the generation and stabilization of non-toxic oligomers [78], probably by consolidating the autoinhibitory effects of the negatively-charged α-Syn C-terminal domain, which stabilizes the oxidized α-Syn, the non-toxic oligomer [78]. In corollary, acetylation of the α-Syn N-terminal domain, which is essential for its plasma membrane binding, has been shown to reduce dopamine metabolite-induced α-Syn oligomerization [79]. Dikiy and Eliezer [80] also reported that N-terminal acetylation increases the α-helicity of the terminal, leading to the stabilization of helically folded α-Syn, which may be critical to the formation of α-Syn tetramer, a native conformation in equilibrium with the α-Syn monomers [81] potentially inhibiting the pathological process of α-Syn oligomerization and aggregation. In similar studies, nitration of α-Syn promotes the formation of highly cytotoxic stable nitrated α-Syn (soluble oligomers), which in turn prevents its transition to fibrils [82], whereas glycation of α-syn in vitro inhibits α-Syn aggregation and produces non-toxic amorphous oligomers [83]. Furthermore, several cell culture and cell-free studies have reported oxidation of α-Syn as the driving force for its oligomerization [84,85]. Qin et al. (2007) [85] reported that the potent oxidant, 4-hydroxy-2-nonenal (HNE), enhanced the stability of soluble oligomers, which inhibited their conversion to fibrils. They concluded that the stable soluble oligomers were highly toxic, confirming the ‘soluble oligomer toxicity theory’. Similarly, monomeric and dimeric nitrated α-Syn have been shown to promote fibril formation, whereas oligomeric nitrated α-Syn inhibited fibril formation by stabilizing the soluble oligomers [86]. Although posttranslational modifications of α-Syn (oxidation-, nitration-, and phosphorylation-induced α-Syn oligomerization) may influence the expression level of α-Syn and have attracted significant research attention as potential factors leading to PD and other synucleinopathies, no simplistic direct pathway to neurodegeneration has been described in the literature, and high expression levels of α-Syn may not be causally related to neuronal dysfunction or death.

Overall, the intrinsically disordered nature of α-Syn is an index of its instability. α-Syn can adopt multiple conformations, including unstructured state in the cytosol, α-helical conformation during membrane binding and β-sheet-rich oligomers and amyloid-like fibrils in Lewy bodies and Lewy neurites as well as non-amyloid amorphous aggregates (neuronal inclusions), which are the pathological hallmarks of PD and other synucleinopathies [87]. It also predisposes α-Syn to several complex interactions with several proteins in neuronal cells, modulating their functions including pro- or anti-apoptotic effects [88]. However, its effect on programmed neuronal cell death has not been reported in greater detail [87,88]. Similarly, α-Syn interacts with presynaptic SNARE to form the α-Syn-SNARE protein complex, which acts as a ‘chaperone’ that controls the folding of proteins, preventing the formation of structured aggregates [89]. Kasen et al. (2022) have reported that monomeric α-Syn inhibited the heat-induced aggregation of truncated 112 amino acid α-Syn, reinforcing its neuroprotective potentials [40]. Thus, any deficiency in α-Syn could lead to protein misfolding and neurodegeneration. α-Syn also regulates synaptic homeostasis and vesicle trafficking, which controls the neuronal synaptic activity as a molecular chaperone by forming the α-Syn-SNARE complexes [44]. These complexes are required for the presynaptic release of neurotransmitters (dopamine) for the control of voluntary and involuntary movements, which might also influence memory and cognitive function [42]. Conversely, the formation of the α-Syn-SNARE complex in a normal physiological solution may lead to the sequestration of arachidonic acid (fatty acid), suggesting an inhibitory effect on neuronal transmission and alteration of lipid signaling. In corollary, polyunsaturated fatty acids (PUFAs) have been reported to enhance oligomerization and neurotoxicity of α-Syn (aberrant effect) [90]. These conflicting findings may be due to the variation in α-Syn expression levels at various stages of PD pathogenesis, which emphasizes the importance of early assessment of α-Syn levels. Therefore, a deeper understanding of the intrinsic structural flexibility and nonintuitive behavior of α-Syn is crucial for identifying valid therapeutic targets in α-Syn-induced PD pathogenesis.

### 2.1. Evidence of Phenotypic Switching and Structure-Toxicity Relationship of α-Synuclein Conformers in PD Pathogenesis

The direct effect of α-Syn conformers in the brain has not been fully described, and their precise conformations have not been structurally elucidated. However, literature is replete with the fact that, under supersaturated conditions, the natively disordered soluble α-Syn monomers undergo spontaneous misfolding and structural transition into highly organized, insoluble, and very stable amyloid conformations enriched with β-sheets (Lewy bodies) through a series of intermediate cytotoxic conformers like oligomers, protofibrils, and mature fibrils within the brain, depending on the presence of certain modulators and other environmental factors [91,92]. Recent reports on the kinetic profiles of α-Syn conformers revealed that the relationship between amyloid oligomer and amyloid fibrils underpins the nature of their cellular toxicity [93]. They reported that low and medium molecular weight intermediate aggregates of α-Syn are metastable and transient but represent the major pathogenic agents in PD; however, they usually dissociate back to monomers rather than forming fibrils [93]. However, the toxic dopamine-induced α-Syn oligomers have recently been shown to be nontoxic [94]. Nonetheless, the loss of the soluble monomeric structure of α-Syn has been reported to coincide with its loss of physiological functions [95]. The authors asserted that aggregation of α-Syn into inclusions like Lewy bodies and Lewy neurites is a normal transition into a more stable state where α-Syn is protected against biological stressors, toxins, and pathogens, reducing the number of normal proteins that are being exposed to danger. It follows that at this stable hibernation state, α-Syn is precluded from having any physiological function or inflicting any toxicity. Thus, Lewy bodies are thought to be nontoxic, and the normal α-Syn monomers do not cause disease, but their conformers are toxic. However, up to now, Lewy bodies, whose main constituent is α-Syn [25], have been described as the hallmark of PD, and the mutant strains of the α-Syn encoding gene, SNCA (A53T), are associated with aggressive forms of PD [24]. Thus, it suffices to state that LB formation exhibits both neuroprotective and neurotoxic characteristics.

In a similar study, Olanow et al. described LBs as “cytoprotective aggresomes” which facilitate the capture and degradation of excess and unwanted proteins through the ubiquitin-proteasome system coupled with the heat shock proteins [96]. It is apparent that there is a quantitative loss of active, functional, soluble, unstable α-Syn protein because of its transition into insoluble, inactive, highly stable cross-β inclusions, and consequently, a loss of physiological functions of α-Syn, which are critical for neuronal survival. Such depleted α-Syn levels have been associated with smaller brain volume and neurotoxicity in PD, where higher levels of α-Syn are required for the preservation of the brain volume [97]. On the other hand, overexpression of α-Syn levels (toxic dose effect) has been associated with more malignant phenotypes [98] because of its propensity to aggregate through nucleation and the consequent reduction of the normal protein levels. The phenomenon of the “toxic dose effect” of α-Syn due to its overexpression has generated some contrasting reports in the literature. Kasuga et al. reported that PD patients with α-Syn SNCA duplication have very low levels of α-Syn in the cerebrospinal fluid [99], reflecting the accumulation of α-Syn in the brain with Lewy pathology. However, Markopoulou et al. tested the hypothesis that genetic overexpression of α-Syn SNCA is associated with worse motor and cognitive function in PD patients and reported that high SNCA expression correlates with better motor and cognitive function compared to low SNCA expression [100]. Furthermore, recent literature is replete with the fact that Lewy pathology in the brain does not correlate directly with the extent of neuronal loss or severity of PD; thus, neither the presence nor distribution of Lewy pathology could predict the motor or cognitive symptoms of PD [101]. Similarly, neuronal loss and cellular dysfunction have been shown to occur prior to α-Syn pathology in the substantia nigra pars compacta [102,103], and the proportion of neurons with Lewy pathology does not correlate with the duration of PD [103].

Furthermore, sufficiently robust correlations of α-Syn strains’ structure with their neuropathological patterns and disease phenotypes in PD pathogenesis and other synucleinopathies are not currently available [20]. These characteristics underpin the α-Syn conformer’s propensity to assume several conformations, and each conformation can spread from neuron to neuron in a “prion-like” manner where they can escape from their host cell, enter into a neighboring neuron via permissive templating [104], and act as a seed for the formation of new aggregates (conformers) using the soluble proteins of the new host neurons [105] which explains the progressive nature of PD. It also predisposes α-Syn to several intermolecular interactions, leading to numerous conformational states that may aggregate or collapse to form several high molecular structures like oligomers and fibrils [66,106], which can be harmful to naïve recipient neuronal cells and cause the spatial spread of pathology associated with the respective phenotypic presentation [107,108]. One of the important mechanisms of α-Syn oligomer-induced cellular toxicity is the disruption of cellular homeostasis by creating a pore in the neuronal membrane, leading to neuronal cell death [109,110]. For example, α-Syn mutants (A30P and A53T) have been reported to form pore-like annular and tubular structures, which have causal links to neuronal membrane permeation and cytotoxicity [111]. In a similar study, Danzer et al. demonstrated that different strains of α-Syn oligomer conformations exert cellular disruption and neuronal death through distinct mechanisms, including cell death via membrane pore formation, while others penetrated the neuronal membrane with enhanced aggregation propensity [110]. In corollary, the accumulation of toxic α-Syn oligomer within the endoplasmic reticulum was directly correlated with the ER stress and PD pathogenesis [112]. α-Syn and its conformers interact directly with the neuronal membranes, membrane-bound vesicles, and receptors, causing nuclear and mitochondrial dysfunction, synaptic impairment, endoplasmic reticulum (ER) oxidative stress, neuroinflammation, neuronal hyperactivity, neuronal death (apoptosis), memory impairment and cognitive deficit [113,114,115]. Several fibril-promoting variants of truncated α-Syn [30,31,32,33,34,35,36,37,38,39,40,41,42,43,44,45,46,47,48,49,50,51,52,53,54,55,56,57,58,59,60,61,62,63,64,65,66,67,68,69,70,71,72,73,74,75,76,77,78,79,80,81,82,83,84,85,86,87,88,89,90,91,92,93,94,95,96,97,98,99,100,101,102,103,104,105,106,107,108,109,110] conformers with amyloid core residues have been reported; however, those containing residues 37–99, which are considered more common polymorph, lack aggregation potential [116,117]. Furthermore, the in vitro human recombinant construct of truncated α-Syn 30–110 conformers produced fibrils more aggressively than the full-length α-Syn in spite of their conformational similarities, whereas in the PD rat model, injection of α-Syn 30–110 fragment exhibited lower toxicity compared to the wild-type full-length α-Syn [118]. Similarly, no significant difference in toxicity of α-Syn 30–110 fragment was observed in human mesencephalic immortalized neuronal cells, compared to the full-length α-Syn [28]. Nonetheless, the full-length α-Syn exhibited a greater extent of cellular dysfunction, indicating that higher fibril-forming conformers may not necessarily correlate to greater toxicity [28]. Therefore, the mechanism of α-Syn conformer toxicity depends on their strain type or species; thus, a deeper understanding of the intracellular activities associated with the α-Syn conformer-induced toxicity is critical to developing clinically effective immunotherapeutic agents for the treatment of PD.

The presence of α-Syn fibrils in postmortem patients’ brain cells has been regarded as the root cause (infectious) of PD [119]. However, several research findings have demonstrated a poor correlation between α-Syn pathology, Lewy body load, and clinical symptoms [120]. Thus, it was hypothesized that soluble oligomers are the potent toxic conformers of α-Syn, not the fibrils [121], indicating that fibrillization alone cannot explain the pathogenesis of PD. It was suggested that the chelation of the α-Syn aggregates and the amyloid fibrils into the inclusion bodies is a protective mechanism by the neuronal cells against the spread of the disease [122]. However, oligomers are regarded as being transient, heterogeneous, dynamic, and metastable, and they are difficult to characterize [27,28]. In corollary, Lindström et al. have demonstrated that the protofibril-specific antibody mAb47 reduced the number of protofibrils and improved motor deficits in mice models [123]. It follows that soluble α-Syn oligomers that do not cause toxicity may exist, and frank inclusions (aggregates) may become neurotoxic over time, highlighting the potential paradigm shift from toxic oligomers to α-Syn fibrils or vice versa. Peelaerts et al. [124] demonstrated the profound differences between the α-Syn conformer strains (oligomers, ribbons, and fibrils) by injecting them into the rat brain. The authors confirmed the striking differences between the strains in terms of strain-specific toxicity, proteolytic cleavage pattern, seeding ability, structural characteristics, and histopathological and behavioral phenotypes. They also confirmed that the α-Syn assemblies crossed the blood–brain barrier and are distributed throughout the CNS. Shahnawaz et al. have utilized conformer-specific fluorescent ligands to differentiate between the conformational states of α-Syn in PD and MSA [125]. The authors also utilized cryo-electron tomography to demonstrate that α-Syn fibrils in PD are straight with less helical twisting compared to the α-Syn fibrils in MSA [125]. In a similar study, Prusiner et al. identified a unique strain of α-Syn prions with a causal link to MSA, which is different from the strain that causes PD or DLB [126], highlighting the fact that different strains of α-Syn are responsible for distinct pathologies and are able to spread in vivo. The heterogeneity of α-Syn conformers is evident from the fact that three clinically and pathologically different neurodegenerative disorders, including PD, multiple system atrophy (MSA), and dementia with Lewy bodies (DLB), are caused by different conformers of the same α-Syn protein [127].

Recently, it was demonstrated that α-Syn can crosstalk with other amyloidogenic proteins like Aβ and tau, which are also associated with Alzheimer’s disease and Huntington’s disease, suggesting that PD progression is associated with other neurological disorders [128,129]. Similarly, α-Syn can crosstalk with bacterial amyloids like CsgA and FapC, which accelerates and inhibits α-Syn expression, respectively, suggesting that amyloidogenic proteins of different origins may influence the onset and progression of PD [130,131]. Furthermore, genome-wide association studies (GWAS) have established a highly significant link between certain single nucleotide polymorphisms (SNPs), which are associated with increased expression of α-synuclein and increased risk of PD [132]. However, overexpression of heat shock proteins (HSPs), which facilitates refolding of aggregation-prone proteins in PD models, and aggregation inhibitors or neutralizers in cellular models of α-Syn overexpression have been shown to confer remarkable protective effects [90,133]. In corollary, Conway et al. (2001) reported the in vitro and in vivo effect of dopamine and its metabolites as inhibitors of the conversion of protofibrils to mature fibrils. They concluded that preferential neuronal vulnerability to the pathogenesis of PD is attributable to the presence of soluble oligomers rather than mature fibril aggregates [134].

Several authors have also reported that the chameleon properties of proteins could foster several pathological conditions. The type of conformation formed by the misfolded α-Syn depends on the neuronal microenvironment; thus, several varieties of conformational strains [135,136] are produced, which are distinct in terms of the incubation period, progression, and type of pathologies that develop in the brain. The amino acids in each conformer undergo specific stretch and intermolecular interactions that lead to structurally and functionally distinct fibril assemblies [137], which have been defined as strains or variants of infectious agents with different incubation periods, clinical symptoms, and extent of histological damage [137,138].

Similarly, it has been demonstrated that agitating human recombinant α-Syn monomers in buffer systems with different salt concentrations produced two different conformations, α-Syn ribbons with flat and twisted shape at low concentrations and α-Syn fibrils with cylindrical shape at physiological concentrations of the buffer salts [139]. Thus, several α-Syn aggregates with different characteristic strains or conformers are deposited in the brain, causing neurodegeneration with different neuropathological and clinical manifestations [91]. It has been suggested that a single precursor wild-type α-Syn with a unique polypeptide sequence can aggregate into multiple conformational strains (conformers) with distinct phenotypic characteristics [91], causing distinct diseases, and that the uniqueness of each α-Syn conformer is due to different structural arrangements of the protein building blocks within the α-Syn aggregates encoding each unique conformer [91]. The conformers have been likened to “infectious isolates” that exhibit distinct prion-like phenotypes, and several studies have buttressed this phenomenon, providing evidence for distinct structures of α-Syn conformers in the brains of individuals with different synucleinopathies [140,141,142,143,144,145]. Yang et al. (2022) utilized cryo-electron microscopy to demonstrate that α-Syn inclusions consist of two types of filaments, each of which consists of two distinct profilaments in the human brain. They concluded that α-Syn filaments (conformers) from the brains of individuals with different synucleinopathies are different and that the distinct conformers are unique to individual synucleinopathy [146]. Furthermore, interconversion of polymorphic states can occur readily; thus, they are present simultaneously in the disease tissue and fibril preparations [91]. Phenotypically different α-Syn conformer aggregates are also referred to as “α-Syn polymorphs” which have different ultrastructural arrangements of the protofilaments with similar protein building blocks [91].

Several techniques have been utilized to unravel the native state of α-Syn conformers, including gel filtration, gradient gel separation, solid-state NMR spectroscopy, cryo-electron microscopy, immunoelectron microscopy, antibody microarray, and mass spectrometry [147,148]. Scanning transmission electron microscopy (STEM)^2^ analysis of α-Syn that was isolated from the fresh human red blood cells and cultured neuroblastoma cells exhibited a natively folded tetramer of several α-Syn molecules of different sizes ranging from several metastable monomers to octamers [81]. In a similar research, Gould et al. demonstrated the presence of conformationally diverse metastable α-Syn multimers in the human brain [149]. They concluded that multimeric conformers of α-Syn are present within the human brain. Recent studies with cryo-electron microscopy and immunoelectron microscopy have revealed the differences between the structural architecture of α-Syn filaments (conformers) in PD patients, which are straight and cylindrical compared to the twisted α-Syn glial cytoplasmic inclusions (GCIs) in multiple system atrophy (MSA) patients [119]. Furthermore, the two types of α-Syn filaments have been detected in MSA patients’ brains, suggesting the co-existence of multiple α-Syn aggregates in the same disease [146]. Thus, further extensive studies would be required to gain clinically useful insight into α-Syn conformational characteristics for the development of in vivo conformation-targeted therapeutic α-Syn conformer inhibitor delivery for the treatment of PD.

### 2.2. Aggregation Propensity of α-Synuclein Conformers in PD Pathogenesis

Under normal physiological conditions, α-Syn monomer exists as a free, soluble, natively unfolded, and unstructured monomer in the cytosol or as a membrane-bound α-helical multimer in equilibrium with the free monomers [43]. It exhibits low hydrophobicity and high charge density [92]; thus, any changes within its microenvironment, like intracellular stresses that increase the hydrophobicity or decrease charge density, may initiate the conversion of the α-helical structure of α-Syn monomers or multimers into toxic aberrant conformers that are rich in amyloid β-sheets. Accumulation of the β-sheets-rich conformers, particularly the large oligomers, disrupts the α-Syn-SNARE complexes leading to cellular stress response, compromised lysosomal functions, and generation of toxic reactive oxygen species (ROS) that cause neurodegeneration and several protein misfolding disorders (PMDs) including PD [150,151]. However, the highly soluble α-Syn monomer is kinetically stable under physiological conditions; thus, the free energy barrier required for α-Syn aggregation is quite high [152], preventing the spontaneous occurrence of aggregation. It follows that the formation of aggregate nuclei is thermodynamically unfavorable under physiological conditions. Nonetheless, changes in the physicochemical properties of the microenvironment may reduce the kinetic barrier, leading to the onset of nucleation at the lag phase and potential acceleration of the aggregation process [153]. When a critical nuclei size is attained, monomeric α-Syn are attracted to the nuclei, enhancing conformer growth at the exponential phase until saturation is achieved and the aggregate is in equilibrium with the free α-Syn monomers at the stationary phase [154]. It is important to note that any addition of prefibrillar seeds into the α-Syn monomers can accelerate the aggregation process by bypassing the lag phase [152].

The α-helical structures of α-Syn have also been associated with its aggregation in the presence of negatively-charged lipids (phospholipids) located on cellular membranes. For example, the synphilin-1 protein promotes α-Syn aggregation and inclusion formation [155]. However, transgenic expression of synphilin-1 in A53T α-Syn transgenic mice reduces neurodegeneration but increases inclusion formation, suggesting that promoting α-Syn inclusion formation is neuroprotective [156]. It is apparent that the native conformations of α-Syn tend to exhibit neuroprotective characteristics in their physiological state. Although the α-Syn complex formation and its transition into aberrant conformers are not easily predictable, high expression levels of α-Syn have been correlated with accelerated aggregation of α-Syn [20], neuroinflammation, and onset of neurodegeneration [40]. Thus, this has prompted intensive research efforts on the development of therapeutic strategies that target α-Syn as a diagnostic biomarker to reduce its levels of expression [157]. However, these research efforts have not yielded any clinically useful disease-modifying therapy so far [158,159]. Furthermore, it is not clear if α-Syn levels are increased in the brains of PD patients, but both increased and decreased levels of α-Syn mRNA in surviving neurons have been reported [160].

**Figure 2:**
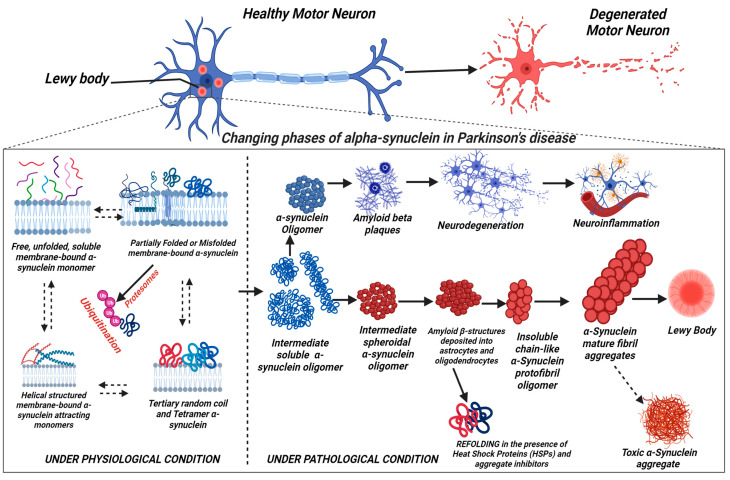
Schematic representation of the aggregation of α-synuclein (α-Syn) in Parkinson’s disease. The unfolded membrane-bound α-Syn is a monomer, which is in equilibrium with the free soluble α-Syn in the physiological solution. Helical structured α-Syn attracts free monomers to for α-Syn aggregates, leading to the formation of oligomers, amyloid sheets, protofibrils, mature fibrils, and Lewy bodies, respectively. Created with BioRender.com (accessed on 30 October 2023).

In corollary, the massive neuronal loss in PD patients has been associated with the conversion of the amphipathic α-helix-rich α-Synuclein protein to a high-order metastable β-sheet-rich aggregate [20,69]. This metastable β-sheet of α-Syn oligomers, like the β-sheet structure of β-amyloid, is deposited into astrocytes and oligodendrocytes where they trigger neurotoxicity and neuroinflammation at molecular, cellular, and organ levels [20]. β-amyloids are self-propagating β-sheet-rich protein aggregates that can coalesce into a tertiary fold in a continuum, leading to the formation of supramolecular stacks called mature fibrils [20]. The process of fibril formation entails the development of misfolded α-Syn oligomeric protofibrils, which are soluble structures initially but gradually become insoluble and coalesce into mature fibrils over time (α-Syn aggregation) [20,161], as depicted in Figure 2. These protofibrils form the pathogenetic basis of the Lewy body and Lewy neurites, which are the hallmarks of PD pathogenesis and other synucleinopathies [161]. It is well documented in the literature that aggregation is the main pathogenic feature of α-Syn because those variants that do not form aggregates (e.g., truncated α-Syn without the NAC domain and the wild-type (WT) β-synuclein) lack neuronal toxicity [133]. Nonetheless, it has been reported that both WT and disease-related mutants of α-Syn (A53T, A30P, and E46K) form amyloid-like fibrils to variable extent when incubated in aqueous solution for a prolonged period [162].

Under pathological conditions, α-Syn loses its native conformation, triggering several genetic mutations, misfolding, truncation, aggregation, and posttranslational modifications (PTM), including the formation of cytotoxic oligomeric and fibrillar conformers and polymorphs that have causal links to neuronal pathology and PD pathogenesis [163]. Interestingly, α-Syn-induced pathology and mutations are also found in the membrane-binding domain, indicating a close association between membrane-binding, physiological function, and pathology of α-Syn. However, the quantitative cause-and-effect relationship between α-Syn expression levels and PD pathogenesis is not clear [163].

The schematic representation of α-Syn aggregation pathways is presented in Figure 2. The free, soluble unfolded monomer has a great affinity for the neuronal membrane; thus, it interacts with the membrane to form unfolded membrane-bound α-Syn complexes, which establish an equilibrium with the free soluble α-Syn in the physiological solution [43]. In pathological conditions, the membrane-bound α-Syn forms an alpha-helical structure, which attracts more free monomers with a high propensity to misfold, mutate, truncate, oligomerize, fibrillate, and accumulate into β-sheet-enriched insoluble conformers within the nigrostriatal neurons, inducing neuroinflammation and neurodegeneration which are the hallmarks of PD pathogenesis [43].

The mechanism of α-Syn aggregation has been described as a three-phase sigmoidal nucleation-dependent polymerization process [161], including the slow lag phase, faster elongation phase, and the stationary phase [161]. Several studies have established that α-Syn aggregation involves the conversion of natively unfolded disordered soluble α-Syn monomer into highly organized amyloid β-sheet-rich α-Syn conformations through a multistep aggregation pathway [162] due to several environment-sensitive conformational transitions [92], including the production of several highly cytotoxic soluble intermediate conformers such as α-helical conformations, oligomers, protofibrils and fibrils [161,164]. These intermediates may appear immediately after the lag phase and remain in the milieu until the mid-exponential (elongation) phase, enhancing and consolidating intermolecular β-sheet formation [153]. It has been reported that the α-helical intermediates consist of oligomeric and prefibrillar conformers that are more cytotoxic than their mature fibrils [68], as discussed in Section 2.1. Nonetheless, the process of aggregation or oligomerization can be controlled by modulating the microenvironmental conditions like the pH, temperature, ionic strength, and α-Syn protein concentration. NMR studies have shown that human α-Syn exhibited a faster rate of aggregation at low pH than at neutral pH [66]. The authors suggested that the partially folded conformation of α-Syn protein is responsible for the faster rate of aggregation at low pH. In a similar study, Wu et al. utilized replica exchange molecular dynamics (REMD) simulations to generate α-Syn conformational ensembles at low and high pHs. They observed a significant structural reorganization at low pH compared to neutral pH in terms of long-range contacts, hydrodynamic radius, and extent of heterogeneity within the conformational structure [165].

Immunoelectron microscopic studies have shown that stained α-Syn Lewy body fibrils are intensely and consistently decorated with α-Syn antibodies in situ [166]. Thus, it was evident that α-Syn is the primary building block of the fibrils of the Lewy bodies, and the insoluble α-Syn filaments accumulate in the brains of PD patients, confirming that the pathological α-Syn may play an important role in the loss of neuronal function [166,167,168,169]. It was noted that the number of cortical Lewy bodies correlates with the severity of dementia and that α-Syn immunoreactive Lewy bodies are the most specific and sensitive marker for dementia in PD patients [14,170,171]. Nonetheless, the Lewy body (LB) is not an obligate biomarker of PD because some familial PD cases do not exhibit the presence of LB.

Furthermore, in PD, dying neuronal cells tend to release aggregated α-Syn conformers, which may interact with endogenous proteins, resulting in the formation of the first low-order assemblies or aggregates, which accumulate rapidly into oligomeric structures followed by higher-order amyloid β-sheet structures through nucleated polymerization mechanism similar to crystal growth [172]. However, there is ample evidence that it is not the protein aggregates themselves that cause neuronal death but the misfolded soluble intermediates like oligomers and protofibrils that are formed during the initial stages of aggregation [28,29,121]. These soluble intermediates are potent neurotoxic species; however, their heterogeneous, transient, and dynamic nature has limited their detailed detection and characterization [27]. Thus, it is not clear whether α-Syn aggregation is a cause or effect of PD pathogenesis because there is no generalized direct correlation between the formation of amyloid-like fibrils and PD.

In general, several in vitro and in vivo studies have indicated that α-Syn misfolding, progressive intraneuronal aggregation, neuroinflammation, several gene (*SNCA*) mutations (A53T, A30P, E46K, H50Q, A53V, A53E, E83Q, and G51D), self-amplification, and gene polymorphisms are significant causal links to the clinical onset and progression of PD [173]. Fayard et al. (2023), in a pilot study, demonstrated for the first time in non-human primates that injection of several strains of α-Syn induced specific functional, morphological, and biochemical alterations, including high aggregation levels of α-Syn in all brain regions and a distinct reduction in the number of neurons in all injected non-human primates which are the features of early but progressive neurodegeneration [174]. Similarly, several studies have demonstrated the role of abnormal filamentous aggregates of the toxic α-Syn in the onset and progression of clinical symptoms and degeneration of the affected part of the brain (brain lesions) in PD. Polymeropoulos et al. identified five Mediterranean families with autosomal dominant PD caused by missense mutation of the α-Syn gene [24], and antibodies specific for α-Syn were used to detect numerous Lewy bodies in sporadic PD and dementia with Lewy bodies (DLB) [15,61,166]. Although only about 15% of PD patients harbor hereditary PD-causing genes, α-Syn’s point *SNCA* mutations, misfolding, accumulation, progressive intracellular aggregation, oligomerization, fibril growth, aggregate migration (cell-to-cell spreading), aggregate amplification, gene polymorphism, and duplications have been extensively implicated in the onset and progression of Parkinson’s disease [11,175,176,177,178,179].

From the foregoing, it is apparent that the α-Syn aggregation pathway has revealed the existence of a heterogenous population of conformers representing a variety of strains with different molecular sizes and structural architecture, where the small oligomers probably provide the nuclei for α-Syn aggregation. In corollary, several extensive research efforts and continuous developments in modern medicine have been made in terms of diagnosis, prevention, and treatment of PD. However, the etiology of PD remains poorly understood, and none of these research approaches has been successfully translated into clinically useful medicines for the treatment of PD. Thus, the main therapeutic strategy has been managing the symptoms of PD, such as movement disorder associated with loss of dopaminergic neurons in the substantia nigra pars compacta. Therefore, a more creative approach is urgently required to target and remove the neurotoxic intermediate oligomeric and prefibrillar conformers with precision and promote intracellular regulation of α-Syn expression levels in cellular and PD models.

## 3. Targeted Degradation and Neuronal Clearance of Aberrant α-Synuclein Conformers in PD Pathogenesis

Generally, any imbalance between the rate of synthesis and biodegradation of α-Syn may lead to the accumulation of misfolded α-Syn conformers and their pathological aggregates, which might interfere with normal cellular processes and propagate neurodegeneration [180]. Thus, proper folding of α-Syn and removal of aberrant proteins through different essential metabolic (proteosome-mediated protein degradation [181]) pathways or cellular recycling process (autophagy [181]) are regulated by the neurons to preserve the cellular homeostasis of α-Syn proteins, which also control their intracellular steady state levels [180,181]. The well-defined pattern of α-Syn pathology in cellular and animal models of α-Syn overexpression has revealed that the deposition of aberrant oligomer and fibrillar conformers of α-Syn is the major driving force for neurotoxicity in PD pathogenesis [182]. Thus, one reasonable option in managing PD may be lowering the α-Syn levels; however, severe reduction (siRNA-mediated downregulation) of α-Syn in adult rats has been reported to cause remarkable neurotoxicity, leading to a 50% loss of nigrostriatal neurons and dopamine concentrations in the rat’s nigral dopaminergic system within 4 weeks, whereas no toxicity was observed when siRNA was injected exogenously at 50–60% downregulation of α-Syn expression [75,183]. It was noted that injecting rat α-Syn into knockdown-aged rats improved their neurodegenerative and behavioral phenotype [184]. Therefore, it is apparent that a certain moderate concentration (expression level) of α-Syn is essential for the survival of neurons in physiological conditions [179], while extremely low and high expression levels may be toxic to the neurons [185]. This nonintuitive behavior of α-Syn would necessitate determining its therapeutic index (safety profile) so that quantitative α-Syn safety levels could be considered for routine diagnostic assay. Another option is to control the rate and extent of α-Syn degradation in neuronal cells. Therefore, promoting controlled α-Syn degradation to prevent a frank surge in its intraneuronal levels or removal of the aberrant toxic α-Syn conformers could provide a template for the prevention and treatment of PD.

**Figure 3:**
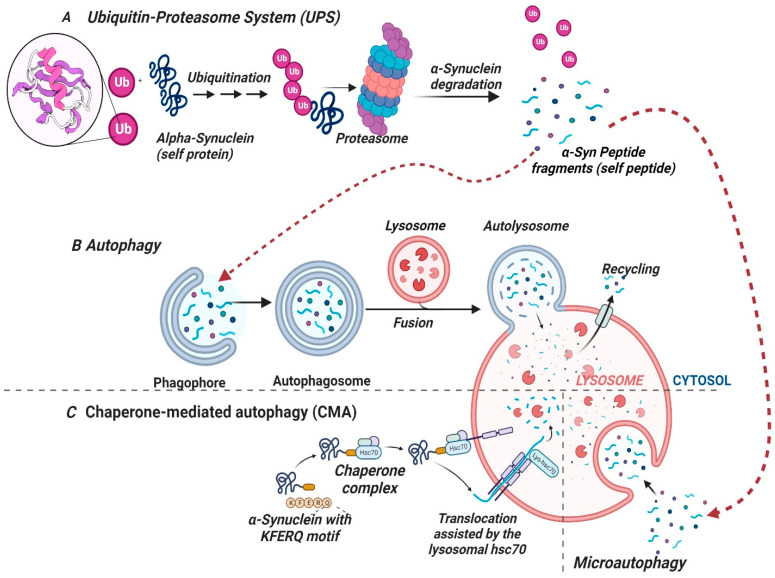
Schematic representation of the degradation of α-synuclein. (**A**) is the hypothetical representation of the ubiquitin-proteasome system (UPS), (**B**) represents autophagy of the α-synuclein fragments and conformers, (**C**) is the chaperone-mediated autophagy and microautophagy. Created with BioRender.com (accessed on 24 December 2023).

Typically, in normal physiological (homeostatic) conditions, misfolded proteins are ubiquitinated, degraded by the ubiquitin-proteosome system (UPS), chaperone-mediated autophagy (CMA) and macroautophagy [186], and eliminated or processed through amino acid recycling as depicted in Figure 3A. However, the ubiquitin-proteosome pathway fails to clear excess α-Syn conformers in PD pathology [187], thus allowing α-Syn misfolding, ubiquitination, and oligomerization without elimination. Therefore, subsequent accumulation of misfolded α-Syn oligomers leads to the formation of pathological α-Syn conformers within the neuron, including oligomers, amyloid β-sheets, protofibrils, mature fibrils, and other polymorphic conformations.

Conversely, defective and redundant cellular components, including aged proteins, damaged proteins, and more complex pathological α-Syn conformers like the aggregates, oligomers, and fibrils are degraded by the autophagy-lysosomal pathway (ALP) to preserve intracellular homeostasis [181]. ALP involves bulk degradation of cytosolic proteins or organelles by forming double membrane cellular structures known as “autophagosomes” which fuse with primary lysosomes to produce “autophagolysosomes” which in turn degrade their contents followed by disposal or further recycling [181] as depicted in Figure 3B. Thus, if the autophagy machinery is faulty, accumulation and aggregation of mutant and toxic α-Syn conformers can occur, which has a causal link to neuronal death in PD pathogenesis. Therefore, regulating autophagy to prevent the accumulation of defective α-Syn conformers in neurons may be a promising neuroprotective approach in the prevention and treatment of PD. However, both degradation pathways may be functionally coupled, where inhibition of one pathway may lead to a compensatory upregulation of the other. Furthermore, any alteration in either of the metabolic pathways may lead to impaired clearance with a consequent accumulation of α-Syn or its conformers. In corollary, overwhelming the pathways may reduce their efficiency and promote the generation of aberrant α-Syn species, leading to neuronal death. As shown in Figure 1 and Figure 3C, α-Syn structure contains a chaperone-mediated autophagy (CMA) recognition motif [VKKDQ (KFERQ-like)] at amino residues 95–99, which allows interaction (binding) with the cytosolic heat shock cognate 70 (Hsc70) and translocation into the lysosome for degradation and recycling via the lysosome-associated membrane receptor protein 2a (LAMP2a). Thus, CMA-based targeted protein degradation has emerged as a therapeutic technique that can selectively target pathogenic proteins and their conformers [188]. Proteolysis-targeting chimera (PROTAC), patented by Kargbo et al., has been utilized for the degradation of wild-type and mutant α-Syn, preventing its aggregation [189]. Most of the degradation of wild-type (WT) α-Syn in neuronal cells occurs through the lysosomal pathways of chaperone-mediated autophagy (CMA) and macroautophagy. Thus, any dysfunction of these pathways may contribute to PD pathogenesis. Aberrant α-Syn oligomers, mutants, and, in some cases, wild-types can induce proteasomal dysfunction, causing CMA impairment and compensatory macroautophagy, which may lead to neurodegeneration.

## 4. Nonintuitive Immunogenicity of α-Syn Conformers in Parkinson’s Disease

Although the extent and mechanism by which α-Syn affects the propagation of PD is not clear, and the deposition of the toxic intermediate α-Syn conformers may be subtle and difficult to detect, the toxic species may trigger cellular and molecular events including immune responses. Thus, this review focuses on the nonintuitive immunogenicity of α-Syn conformers and their neuro-immunotherapeutic potentials. It is envisioned that the subtle but toxic α-Syn conformers could be valid therapeutic targets for the development of potent and clinically effective immunotherapeutic agents for the prevention and treatment of PD.

Recently, investigations on the native functions of α-Syn in healthy neurons have attracted significant research interest to unravel the potential influence of α-Syn on the immune response [190]. The α-Syn protein has been shown to be the major PD biomarker [191]. However, immune responses to the various toxic α-Syn conformers, including oligomers, protofibrils, and their soluble and insoluble aggregates or polymers, which are also potential biomarkers in PD, have received little research attention to date. It is envisioned that understanding the nonintuitive immunogenicity of toxic α-Syn conformers could provide insight into immunotherapeutic targets for more effective prevention and treatment of PD and other neurodegenerative disorders. Although the immune system typically fights against foreign antigens, not self-antigens (self-tolerance), it is well documented that it protects PD patients from toxic α-Syn self-antigens through autoimmune reactions that can be triggered in PD pathogenesis [192]. Autoimmunity occurs through several mechanisms, including genetic alteration of the pattern recognition receptors (PRRs), cross-reaction of the host immune cells with α-Syn conformer self-antigens (molecular mimicry), antigenic epitope spreading and T cells and B cells dysfunction [193,194]. α-Syn neurotoxicity has largely been attributed to its soluble and insoluble aberrant intermediate conformer strains and their aggregates, which act as the neuropathological hallmark in PD patients’ brains [194], inducing high neurotoxicity via inflammation, oxidative stress, and autophagy dysfunction [195,196]. The actions of α-Syn and its conformers as self-antigen in the PD autoimmune process were documented by Benner et al., who reported that immunization of effector T cells by nitrated α-Syn, a self-antigen, exacerbated neuroinflammation and increased neurodegeneration in PD mouse model [197]. Thus, nitrated-α-Syn and other toxic posttranslationally modified conformers could act as self-antigens and biomarkers for the clinical diagnosis of PD, breaking down the hosts’ immunological tolerance and inducing autoimmune responses that could exacerbate PD pathogenesis. In corollary, Cao et al. demonstrated that overexpression of α-Syn plays a critical role in PD pathogenesis by triggering microglial activation and adaptive immune responses leading to neurodegeneration [198]. The authors selectively overexpressed human α-Syn by injecting adeno-associated virus serotype 2 (AAV2) into the substantia nigra of wild-type mice and Fc gamma receptor negative (Fc-γR-/-) mice which lack an affinity for IgG. They demonstrated that overexpression of α-Syn led to the expression of a specific antigen that induces the generation of immunoglobulin G (IgG) and the expression of the Fc-γ receptor on the microglia membrane that binds the IgG. These expressions led to strong microglial activation, expression of proinflammatory molecules, and degeneration of dopaminergic neurons in the wild-type mice, triggering humoral adaptive immune responses, whereas microglial activation was attenuated in Fc-γR-/- mice, which were protected from α-Syn-induced neurodegeneration [198]. Therefore, α-Syn is considered critical to the induction of autoimmune responses that precede neurodegeneration.

### 4.1. α-Syn Conformer-Induced Immunogenic Response in PD Pathogenesis

Research results have shown that α-Syn is abundantly expressed in the CNS, including the cerebral cortex, thalamus, neocortex, hippocampus, substantial nigra, and midbrain, as well as the brain’s immune cells, including glial cells [40,90,199]. Thus, several therapeutic approaches to mitigating the signs and symptoms of PD have focused on regenerative stem cell replacement and intracellular clearance of α-Syn. However, these approaches have not shown any long-term clinical benefits. Baglioni et al. have reported that α-Syn prefibrillar aggregates and other amyloid species are generic toxins [200], which was also evident as increased levels of α-Syn oligomers in PD patients’ plasma in an in vivo study conducted by El-Agnaf et al. [201], confirming the in vivo transformation of α-Syn into toxic oligomers in PD pathogenesis. It is common knowledge that α-Syn aggregation and interneural propagation contribute to PD pathogenesis [202]. However, RNA interference has been utilized to inhibit α-Syn expression in PD patients [203]. Similarly, α-Syn overexpression in the extracellular environment can be recognized by specific α-Syn antibodies and cleared by macrophages to prevent further interneural propagation [204]. Thus, it is apparent that α-Syn strains’ cellular binding, uptake, accumulation (clearance resistance), and strain-specific seeding may trigger inflammatory and immune responses, and tracking such immunogenic responses would be of great clinical and scientific value. In familial PD, the multiplicity of α-Syn gene SNCA may lead to an increase in its expression levels [205], while in other circumstances, SNCA mutations predispose α-Syn to spontaneous aggregation [175]. However, there are no therapeutic agents targeting all the pathologic aberrant α-Syn conformers [190], including oligomers, protofibrils, fibrils, truncated α-Syn, α-Syn polymorphs, and strains, as well as posttranslationally modified α-Syn conformers. Therefore, a more creative multipronged neuroprotective approach is required to control the immunogenicity of neurotoxic α-Syn conformers, develop precision-driven target identification and validation techniques, develop novel specific toxic α-Syn conformer-targeted immunotherapeutic nanocarrier delivery systems, control the α-Syn conformers endogenous recycling process, and restore the brain’s microenvironment homeostasis through controlled neuroimmune responses. The α-Syn cascade hypothesis indicates that α-Syn aggregates are formed long before the manifestation of clinical symptoms in PD [115]. Thus, two sequential phases of α-Syn pathology are apparent: the formation and accumulation of the intraneuronal inclusions (Lewy bodies) in the nigrostriatal system and direct or indirect neurodegeneration [115]. It is apparent that α-Syn and its conformers may penetrate the neuronal membranes, causing neuronal damage (primary lesion), membrane leakage, neuronal lysis, and subsequent release of neuronal contents into the brain’s microenvironment. Thus, active immunotherapy or the formation of antigen-antibody complexes (passive immunotherapy) could promote clearance of aberrant α-Syn via lysosomes and microglia [190]. Presently, α-Syn-specific antibodies are utilized in the examination of α-Syn aggregates in human postmortem brain tissues by targeting epitopes within the C-terminus, N-terminus, and NAC domains of the α-Syn [72]. Thus, profiling the immunogenicity of α-Syn conformers as potential biomarkers for PD should be achievable. However, emerging research evidence suggests that C-terminally truncated α-Syn strains exhibit a significantly high propensity to aggregate and cause neurotoxicity [206,207,208]. Thus, many α-Syn-specific antibodies fail to capture the toxic strains [72]. Zhang et al. reported the C-terminus truncation-induced alteration of the α-Syn into elongated conformations by disrupting the long-range intramolecular interactions between the C- and N-terminal domains [208], which increased the exposure of NAC and the N-terminus and resulted in stronger interaction of the C-terminus truncated α-Syn with cellular membranes and a molecular chaperone with consequent mitochondrial dysfunction [208]. The inherent complexity and remarkable chameleonic structural plasticity of posttranslational modified (PTM) and truncated variants of α-Syn enhanced its ability to aggregate and adopt myriads of tertiary conformations [92], which presented multiple epitopes in the same sample, and certain epitopes may be masked, preventing interaction with the antibodies. Nonetheless, despite the inherent complexity of α-Syn structural architecture and its conformational diversity in the human brain, several authors have studied the staining profiles of posttranslational modified- and epitope-specific α-Syn antibodies using single-epitope immunolabelling techniques [209,210]. Kovacs et al. have demonstrated the strong affinity of the N-terminus antibody (5G4) for the high molecular weight fraction of β-sheet-rich α-Syn oligomer, facilitating the preferential immunolabelling of the pathological α-Syn conformer [211]. Altay et al. demonstrated the detection of astrocytic α-Syn conformers within the human brain by unlabelled antibodies directed against the mid-late N-terminus (LASH-BL 34–45, 5G4) and late NAC domain (LASH-BL 80–96) [212]. Similarly, Moors et al. utilized PTM- and epitope-specific α-Syn antibodies to characterize the structural arrangement of several α-Syn strains within the Lewy bodies [213].

**Figure 4:**
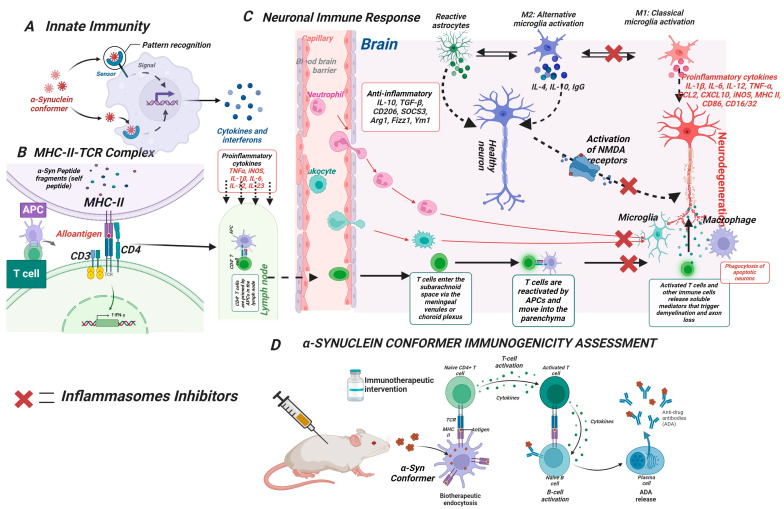
Schematic representation of immune response of α-synuclein (α-Syn) in Parkinson’s disease. (**A**) Innate immune response, (**B**) MHC-II-T cell receptor complex, (**C**) neuronal immune response to alpha-synuclein conformers, (**D**) alpha-synuclein conformers immunogenicity assessment. Created with BioRender.com (accessed on 24 December 2023).

In corollary, a neuronal injury could trigger the genetically encoded ‘response to injury wound healing (RTIWH) mechanism’, which consists of four phases: homeostasis, inflammation, proliferation, and remodeling followed by repair and resolution if the injurious stimulus is removed [214]. However, α-Syn conformers (oligomers and fibrils) are chronic injurious stimuli that are not easily removed; therefore, they cause persistent response to injury wound healing with remodeling but no resolution. The microglia reactive astrocytes (CNS innate immune system), which is the cell type responsible for scarring in the brain, will interact with leukocytes (macrophages, monocytes) recruited from the peripheral system and activate the production of proinflammatory cytokines (inflammatory responses) to neutralize and eliminate the debris, damaged cells, and abnormal α-Syn aggregates from the extracellular space as depicted in Figure 4. It is well documented that the major paradigm for PD pathogenesis is the accumulation of abnormal aggregate of the protein α-Syn in neural and glia cells, resulting in the formation of intraneuronal inclusions called Lewy body, which has been associated with neurotoxicity in the brain. However, 8–15% of autopsies done on people who did not manifest neurological symptoms exhibited α-Syn aggregation in the brain (incidental Lewy bodies) [40], indicating that aggregation of α-Syn alone cannot fully explain neurodegeneration in PD. Although Lewy bodies are considered relatively nontoxic, they are prone to the formation of soluble metastable α-Syn conformers (oligomers and fibrillar structures), which are neurotoxic [40]. Thus, we posit that the soluble metastable α-Syn conformers, which have not been quantitatively evaluated in literature to date, might constitute soluble antigens that trigger neuronal immune responses and could be better therapeutic targets for the treatment of PD. In corollary, it has been reported that the extent of PD severity is α-Syn dose-dependent [215]. Thus, increasing doses of α-Syn lead to α-Syn aggregation, resulting in its loss of physiological function when physiologically active α-Syn monomers are sequestered into the pathological aggregates (conformers), thereby leading to α-Syn dysfunction at the molecular, cellular, and organ levels. The increasing doses also result in gain-of-neurotoxicity function, triggering α-Syn pathology and PD pathogenesis [215]. However, it is not clear how much of α-Syn is needed to maintain its physiological functions at the synapse (α-Syn synaptic level) or whether the toxic effects are independent of its physiological function.

Cellular expression levels of α-Syn have been reported to increase during bacterial and viral infections, particularly during the recruitment of immune cells and lymphocyte maturation, suggesting its critical role in pathogen-activated immune response [40]. Therefore, there is a renewed interest in α-Syn directed immunotherapy for PD. It has been reported that the cells of innate and adaptive immune systems are found in the choroid plexus, meninges, and perivascular spaces of the brain, known as immunological niches, that regulate leucocyte homing to the brain parenchyma when required [216]. It suffices to state that the CNS has a sufficiently robust and resilient self-protective and tightly controlled immunological network for the maintenance and repair of its tissues. Thus, it is important to understand which element of the brain’s immunological network becomes dysfunctional during α-Syn pathology in PD.

Similarly, the cell-to-cell transmission of the misfolded aberrant α-Syn released from dead or damaged neurons into the extraneuronal microenvironment is likely to trigger activation of the microglia, the brain’s resident immune cell, to migrate (chemotaxis) toward the site of damage (inflammation) and release proinflammatory cytokines and chemokines like tumor necrosis factor-α (TNF-α), interleukin-1β (IL-1β), interlukin-6 (IL-6), interleukin-8 (IL-8) and interferon-γ (IF-γ) which are neurotoxic factors, as depicted in Figure 4A (innate immune responses). The proinflammatory cytokines are transported to the lymph nodes where they activate antigen-presenting cells (APCs) like dendritic cells and trigger the generation of major histocompatibility complex class II (MHC-II) molecules which can cross the blood–brain barrier (BBB) to present the epitopes of aberrant α-Syn conformer peptides to the autoreactive T cells for destruction (Figure 4B,C). MHC-I molecules can also present α-Syn peptide antigen directly to the T cells for destruction. Although T cells of healthy people can recognize self-antigens from the misfolded α-Syn or its conformers, only a few antigen-recognizing regulatory T cells (Treg) are found in PD patients. Thus, α-Syn peptide antigen may escape the immunological tolerance, leading to a severe autoimmune reaction. Therefore, we posit that the easiest immunotherapeutic targets would be to prevent or attenuate neuroinflammation, as indicated by the “crossed” symbol (**X**) in Figure 4C, and reestablish immunological tolerance, which is the functional role of the Treg. Literature is replete with the fact that effector and regulatory T cells can be neurotoxic or neuroprotective in controlling the brain’s microenvironment; however, the mechanisms of their immune responses are still being studied. Helper T cells can activate the production of anti-α-Syn antibodies outside the brain, which can pass through the blood–brain barrier and bind with the α-Syn epitopes on the neurons to activate the complement system and initiate antibody-mediated cytotoxicity. Reports of phagocytosis of α-Syn by microglia are also available in the literature; however, it is not clear if α-Syn can directly activate microglia and macrophages by itself. Thus, the aberrant α-Syn conformer could be a prime therapeutic target for the development of novel and potent immunotherapeutic agents for the prevention and treatment of PD.

Figure 4D shows the schematic representation of the immunogenicity assessment for the α-Syn conformers. Aggregation and conformer formation are potential risks for unwanted immunogenicity of biotherapeutics like α-Syn, which can compromise product efficacy and trigger potentially serious adverse effects. However, this process could be utilized to generate anti-α-Syn conformer antibodies in patients to eliminate the aberrant α-Syn conformers. As shown in Figure 4D, administration of α-Syn biotherapeutics containing its conformers may induce the generation of MHC-II molecules, the antigen-presenting cells that bind to and display the α-Syn peptide antigen (epitopes), followed by forming a complex with the naïve CD4^+^ T cells (helper T cells). The helper T cell is then reactivated to produce cytokines that activate the plasma cells to produce specific anti-α-Syn antibodies. It has been reported that the α-Syn with the highest order of aggregation is the most immunogenic [217]. All aggregate sizes reported in literature ranging from 0.1–100 µm had a significant effect on the immunogenicity of α-Syn [218]. However, some low-order-level aggregates may exhibit greater immunogenicity, and misfolded α-Syn may exhibit different epitopes (foreign) with a risk of losing self-tolerance. Furthermore, posttranslational modifications like oxidation have been found to be highly immunogenic, while the effects of phosphorylation have not been widely studied.

In general, immunogenicity is evaluated by subjecting biotherapeutics or existing therapeutic proteins like monoclonal antibodies to quantifiable stress like heat, chemical reaction (oxidation), and agitation or stirring followed by in vitro or in vivo assessment of their immunogenicity potential. However, the heterogeneity of α-Syn conformers is a huge challenge as it is difficult to disentangle the specific aggregate characteristics that directly impacted the immunogenicity index. Therefore, a robust and systematic approach is needed where the detailed mechanism of aggregation is elucidated, and each aggregate characteristic is controlled and precisely mapped to specific immunogenicity endpoints.

### 4.2. α-Synuclein-Induced Inflammation in PD Pathogenesis

Although the mechanism of α-Syn aggregation and how it causes neurodegeneration in PD is not fully understood, neuroinflammation is increasingly being associated with PD pathogenesis. Thus, many research efforts are focused on markers of microglial activation and quantification of inflammatory cytokines in cerebrospinal fluid (CSF), plasma, and the PD brain. Elevated levels of cytokines like TNF-α, IL-1β, IL-2, IL-4, IL-6, and transforming growth factor-beta 1 (TGF-β1) have been identified in the substantia nigra and striatum of the PD patients’ brains [219]. However, current research reports have not been able to distinguish between α-Syn-induced inflammation and neurodegeneration-induced inflammation, as both can occur simultaneously. Thus, intensive research has been focused on markers of microglia activation and quantification of microglial-associated inflammatory cytokines in PD brain, cerebrospinal fluid (CSF), and plasma. Recently, the role of oligomeric α-Syn aggregate in initiating glial and neuronal inflammation via activation of several proinflammatory components has been reported in the literature [220,221]. α-Syn conformers, oligomers, protofibrils, and fibrils have been shown to activate the nucleotide-binding oligomerization domain containing protein 3 and leucine-rich repeat-containing receptors (NLRP3) inflammasome, which is the first line of defense against non-self-pathogens, indicating an innate immune response at the beginning of PD pathogenesis. The hyperactive inflammasome senses danger and responds by activating the gene that encodes interleukin-1β (IL-1β), leading to a self-sustaining inflammation, which is a protective response to cellular or tissue injury, bacterial-, viral-, and fungal infection, and pathogen-free inflammation. However, it can cause severe damage to healthy cells and tissues. Inflammatory response increases blood flow to the site of infection or injury, leading to accumulation and activation of leukocytes like neutrophils, macrophages, Langerhans cells, and dendritic cells to eliminate the pathogen. It also activates a cellular self-destructive sequence leading to cell death, which is known as pyroptosis. Thus, NLRP3 inflammasomes are linked to several chronic inflammatory diseases like inflammatory bowel disease and PD. One therapeutic strategy to slow or stop the progression of these diseases is to administer inflammasome inhibitors like hydrocortisone, prednisolone, and dexamethasone, as indicated by the symbol “X” in Figure 4C; however, their wide range of side effects limits their effective clinical use. Thus, there is an intensive search for inflammasome inhibitors with better safety profiles. Similarly, clinical research using PD animal models has been very challenging. Many studies have evaluated neuroinflammation resulting from α-Syn-induced neurodegeneration using neurotoxicant models and transgenic α-Syn overexpression human models. However, it has been very difficult to model the prolonged period of α-Syn aggregation preceding the dopaminergic neuron loss. The neurotoxicant models produced neurodegeneration without α-Syn pathology, while transgenic models elicited significant α-Syn pathology with little or no significant neurodegeneration. It is apparent that α-Syn overexpression models do not take cognizance of the intrinsic nonintuitive characteristics of α-Syn conformers at normal endogenous expression levels of α-Syn. Therefore, research in this area is continuous.

## 5. Strategies for Immunotherapeutic Delivery Intervention in PD

Presently, the approved therapeutic agents used in the temporary control of clinical symptoms of PD include levodopa/carbidopa, dopamine agonists, monoamine oxidase-B (MAO-B) inhibitors, catechol-O-methyltransferase (COMT) inhibitors, anticholinergics, and amantadine. However, these medications are not able to cure the disease [222]. Furthermore, long-term use of these medications leads to tapering efficacy and potentially serious adverse side effects. Therefore, identifying specific drug targets for the development of clinically effective disease-modifying therapeutic agents that will prevent the progression of PD and other neurodegenerative diseases is still an unmet clinical need. Thus, the prevalence of neurodegenerative diseases is increasing rapidly. It has been reported that by the time a patient manifests the symptoms of PD, approximately 90% of dopaminergic neurons have been lost [223]. Therefore, there is an urgent need for creative approaches to early therapeutic intervention that will prevent further loss of neurons and prevent the formation and transmission of aberrant α-Syn conformers. This could be a template for unraveling the biochemical causes of α-Syn aggregation and conformer formation and for developing effective diagnosis, prevention, and disease-modifying strategies for the treatment of Parkinson’s disease.

### 5.1. Targeting α-Syn Uptake Receptors

The α-Syn cascade hypothesis suggests that the membrane-bound α-helical α-Syn multimers attract monomers and accumulate to form the self-propagating fibrils, which bind to the neuronal surface and penetrate the neuron; however, this may be cell-type selective. The uptake and internalization of the fibrils depend on specific surface receptors and transmembrane glycoproteins like heparin sulfate proteoglycans (HSPGs). Heparin is an α-Syn uptake inhibitor that competes with HSPGs to bind with the neuronal cell surface and prevents the internalization and spreading of α-Syn fibrils [224]. It was noted that some surface receptors bind to α-Syn fibrils but not oligomers or monomers. It is interesting to note that activated microglia and T cells, which are critical to PD pathogenesis, play a significant role in the propagation of α-Syn seeds, emphasizing the significance of immunotherapeutic strategies for PD.

### 5.2. Targeting Total α-Syn Expression Levels

Several studies have used α-Syn expression levels as biomarkers for PD and other synucleinopathies because of their significant importance in PD pathogenesis. Most studies reported a reduction of total α-Syn levels in the cerebrospinal fluid (CSF) of PD patients compared with control groups [225]. In contrast, the level of oligomeric α-Syn was higher in PD patients compared with the control group [226]. Therefore, the data from α-Syn measurements in peripheral tissues and fluids have been inconsistent. Thus, peripheral α-Syn levels do not seem to have good potential as biomarkers for PD. We hypothesize that drug molecules or other therapeutic agents that can modulate the protofibril intermediates of α-Syn, render them non-pathogenic, and remove them from the neuronal cells would be a good therapeutic strategy for the treatment of PD.

Therapeutic strategies targeting α-Syn are still being implemented by reducing its level via either RNA interference or using antisense oligonucleotides. However, α-Syn may exhibit nonintuitive opposing effects in the brain, promoting neurogenesis and neuronal maturation (neuroprotective) [227]. Thus, targeting the monomeric form of α-Syn directly might inhibit its normal physiological functions, which may lead to neuronal death. Therefore, the therapeutic index of α-Syn should be carefully determined to avoid excessive reduction in its expression level such that PD progression is controlled without interfering with its physiological functions. Therefore, specifically targeting the oligomers and fibril conformers may be the most effective approach to preventing PD pathogenesis without unwanted side effects. Furthermore, α-Syn exerts neurotoxic effects on the neighboring neuronal cells, particularly at higher concentrations (overexpression). Therefore, the development of α-Syn-targeted therapies needs to reconcile these nonintuitive characteristics.

Immunotherapy against intracellular α-Syn in transgenic mice has been shown to decrease protein aggregation and neurodegeneration. For example, Tran et al. (2014) demonstrated that α-Syn monoclonal antibodies (Syn211 and Syn303) blocked the entry and cell-to-cell transmission of aberrant α-Syn (oligomers, preformed fibrils) in primary neurons, which prevented the propagation of α-Syn pathology to other neurons [21,23,228]. However, the mechanism by which passive immunotherapy inhibits cell-to-cell transmission of aberrant α-Syn is unknown.

Recently, targeted protein degradation (TPD) has emerged as an immunotherapeutic tool for neurodegenerative diseases including PD. It utilizes protein degradation technologies, including proteolysis-targeting chimeras (PROTACs), molecular glue, and hydrophobic tagging in which the target protein is degraded through intracellular proteolytic machinery, including proteasomes and lysosomes (Section 2). This tool is precision-driven to regulate protein levels and maintain in vivo therapeutic efficacy concurrently; however, it is presently focused on cancer immunotherapy but has potential for application to other therapeutic areas including PD.

### 5.3. Targeting α-Syn Conformers

Literature is replete with the fact that α-Syn aggregate conformers are the hallmarks of the onset and progression of PD pathogenesis. Large aggregates have the potential to fragment within the neuronal cell to form new nuclei for the propagation of α-Syn conformers from one neuron to another. However, since the aggregates progressively increased in size and complexity, and fragmentation may lead to the formation of several polymorphic conformers, it is not clear whether the aberrant α-Syn released by the injured neuron is the intact large aggregates or the fragmented conformers, which conformer penetrated the neuronal membrane, and which one is the most toxic. Gao and fellow scientists have demonstrated that Human Hsp70 disaggregase enzyme fragmented α-Syn fibrillar aggregates into α-Syn monomers and oligomer seeds that can move from neuron to neuron, thus reversing the α-Syn fibrils-induced aggregation in PD [229]. The proteases (disaggregases) in the lysosome could also fragment the α-Syn fibrils, releasing smaller conformers as seeds, particularly at low pH. Thus, the use of aggregation inhibitors is a promising strategy to prevent aggregation of α-Syn and a potential therapeutic target. Research is ongoing to characterize the specific α-Syn conformers produced through protein disaggregation pathways and their relative degree of propagation. Targeting cellular clearance of misfolded α-Syn species using immunotherapies is also a viable approach to preventing its aggregation and formation of oligomers and fibrils. Thus, genetic modifiers of misfolding, oligomerization, and fibrillization mechanisms could be the blockbuster change in PD pharmacotherapy [5].

One of the greatest challenges of therapeutic intervention in PD pathogenesis is the α-Syn conformational switch from a nontoxic, disordered monomeric species or α-helical multimer to the aberrant insoluble β-sheets-rich α-Syn oligomers and fibrils with several distinct polymorphs or conformers that are the hallmarks of α-Syn pathology. More research evidence is emerging from the genome-wide association studies (GWAS) indicating the existence of several α-Syn conformers, each strain with its unique biochemical characteristics that are responsible for distinct clinical manifestations of PD symptoms. Thus, it is essential to understand the triggers and mechanisms by which the nontoxic soluble monomeric α-Syn is converted to the toxic insoluble aberrant β-sheets-rich α-Syn fibrils. In vitro experiments have shown that increased concentration of α-Syn increased the rate of fibrillization, which may be similar to the in vivo results where high expression levels of α-Syn increase the propensity of forming α-Syn inclusions. It would be interesting to investigate the neurotoxic capacity of the different α-Syn conformers and whether a ‘one-for-all’ or ‘fixed-dose combination’ therapy would be more effective for the treatment of multiple strains in the near future. Oligomers and protofibril conformers have been particularly difficult to model because they are quite soluble when freshly formed and inherently transient, rapidly attracting free α-Syn monomers to form the insoluble mature fibrils. Therefore, developing specialized biomarkers for monitoring α-Syn biochemical activities, molecular factors that regulate the polymorphic transition and expression thresholds in the brain microenvironment and designing customized immunotherapeutics that can cross the blood–brain barrier effectively with suitable pharmacokinetic profiles and therapeutic window, and target specific α-Syn conformers would be of great clinical and scientific interest. Furthermore, because of the heterogeneity of the α-Syn conformers, it is possible that there is more than one transmission pathway such that blocking one pathway may not be effective as other pathways may be activated to compensate for the blockage. Thus, a multipronged approach targeting two or more pathways would be of great value.

### 5.4. Targeting Antigen-Presenting Cells (APCs)

Microglia, the dendritic cells (DC) of the brain, are important immune regulators, presenting antigens (epitopes) to the macrophages and T cells, and they have significant expression of α-Syn. DC has a high expression of MHC-II molecules and is equipped to detect and respond to damage-associated molecular patterns (DAMP) in the brain. It is well documented in the literature that DCs can migrate to the CNS during development and in the presence of inflammation. The presence of α-Syn monomers and oligomers aggregates activates the maturation of DC. However, the mechanism of maturation remains unclear. As described in Section 4 (Figure 4), the CD4^+^ T cells are activated to become the effector cells in response to the α-Syn conformer antigen presented by the MHC-II molecules, while CD8^+^ T cells become the cytotoxic T cells that kill the infected or damaged cells. The significance of targeting APCs is that T cells can form memory cells against α-Syn conformers, and the degree of neurotoxicity can be predicted by the population of CD4^+^ T cells. For example, depletion of the CD4^+^ T cell population indicates neuroprotection. Similarly, a decrease in the total T cell population and lower CD4^+^ T/CD8^+^ T ratio indicate neuroprotection.

### 5.5. Targeting α-Syn Inflammatory-Related Biomarkers for PD

Neuroinflammation is a critical hallmark of PD, and a deeper understanding of the impact of neuroinflammatory mediators on the pathogenesis of PD could spark some advancement in the treatment of PD. α-Syn oligomer aggregates have been reported to activate microglia in PD mouse models, releasing reactive oxygen species (ROS), which may exacerbate neuroinflammation and PD pathology. Thus, anti-inflammatory therapy is a crucial intervention strategy to prevent or slow down neurodegeneration and PD pathogenesis, as discussed in Section 4.1. Since the inflammatory response could occur slowly for several decades before the manifestation of clinical symptoms of PD in patients, identification of early neuroinflammatory biomarkers for PD should be a priority. However, it may be difficult to create such a timeline in animal models. Furthermore, most of the biomarkers (cytokines) are difficult to detect or quantify in the patients’ plasma, and most of them are non-specific. Therefore, a combination of biomarkers may be necessary or identifying specific functional biomarkers that encode the functional status of the inflammatory cells before any damage to the neurons.

### 5.6. Harnessing Smart Drug Delivery Systems for Immunotherapeutics in PD

Despite the extensive research into promising therapies for over a century and continuous development in modern medicines, there is still a lack of clinically effective disease-modifying drugs or immunotherapeutic agents for the treatment or prevention of PD and other neurodegenerative diseases. Most of the challenges impeding the translation of fundamental research into bedside therapies include diverse mechanisms of neurodegeneration, multiple concurrent triggering factors, multifactorial onset of symptoms, defective protein degradation machinery, nonintuitive α-Syn protein dynamics providing extensive range of therapeutic targets, free radical formation, impaired bioenergetics, oxidative stress, heavy metal toxicity, and mitochondrial dysfunction [230]. The emergence of several innovative therapeutic technologies like stem cell therapy, targeted protein degradation techniques, microbiome intervention technology, and gene silencing techniques is a beacon of great hope that clinically effective immunotherapy will be available very soon. However, the current near-zero success rate demands urgent concerted research efforts to foster innovative advancement in rational drug delivery strategies by utilizing novel precision-driven informatics, quality by design, predictive mappings, automation, process analytical nanotechnology, and artificial intelligence to understand the nonintuitive behavior of α-Syn conformers at the molecular level and develop a library of immunotherapeutic agents with well-defined architecture, novel surface characteristics and unique mode of action to enhance their potency, endogenous stability, PK profiles, bioavailability, and therapeutic efficacy.

Presently, the route of administration for immunotherapeutics is largely parenteral. Thus, the rational formulation design of smart drug delivery systems for native and therapeutic proteins could provide great delivery efficiency and diversity, as well as regulation of protein functions and stability. The immunotherapeutic approach entails the administration of active or passive vaccines against aberrant α-Syn, which increases the intracellular clearance of the toxic conformers by autophagy (Figure 2) or macrophage activation (Figure 4) and promotes neuroprotection. Effective immunization must target specific conformers or strains to prevent unwanted side effects and autoimmune inflammatory responses. It is important to note that immunization might interact with endogenous native proteins, exacerbate neuroinflammation, and induce autoimmunity. Thus, a rational formulation design of vaccines is required to minimize the adverse effects. Masliah et al. (2005) [21] demonstrated the effectiveness of active immunization against α-Syn using a recombinant human α-Syn in a transgenic mouse model. They reported the production of antibodies with higher affinity for the recombinant human α-Syn and decreased accumulation of α-Syn in the neuronal cell bodies and synapses with the consequent reduced neurodegeneration. They concluded that active immunization against α-Syn did not trigger neuroinflammatory responses in the mice. It suffices to state that preventative immunization may be more effective and presents fewer side effects than therapeutic immunization, particularly before the buildup of the intraneuronal α-Syn aggregates (onset of PD). Passive immunization facilitates clearance of the toxic α-Syn aggregates by administration of exogenous antibodies against the human α-Syn. Masliah et al. (2011) [23] demonstrated that the antibodies recognized and bind with the different domains of the α-Syn, including the N-terminal and C-terminal epitopes (Figure 1). They concluded that passive immunization can be of great therapeutic value in the management of α-synucleinopathies, as the antibodies can cross the blood–brain barrier, form antigen-antibody complexes that are cleared by endocytosis and transported to the lysosomes for degradation via autophagy.

Examples of immunotherapeutics targeted against α-Syn in PD clinical trials are presented in Table 1 [37,231,232].

#### Nanocarriers for Immunotherapeutic Brain Delivery

**Figure 5:**
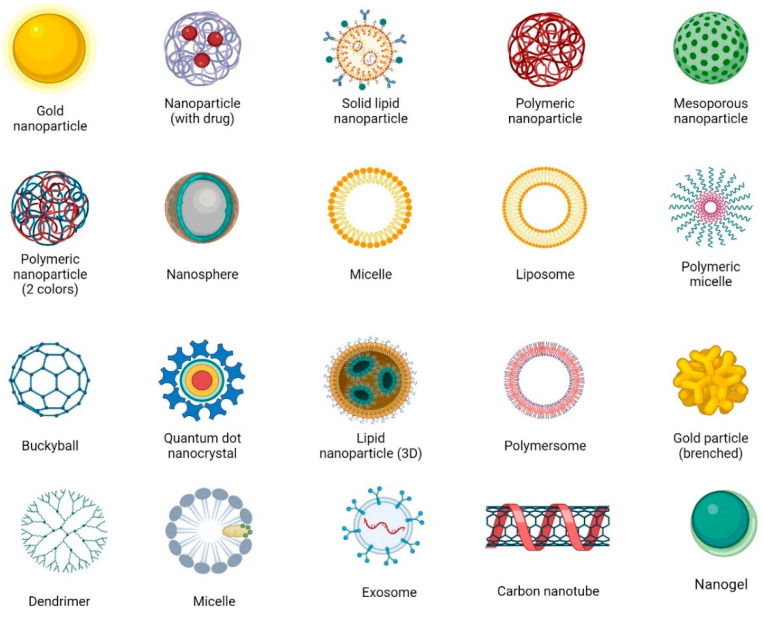
Schematic representation of nanoparticle-based immunotherapeutic brain delivery in reference to Table 2. Created with BioRender.com (accessed on 31 December 2023).

The blood–brain barrier (BBB) is the toughest protective membrane, with several tight junctions that prevent virtually any drug molecule from passing through to the brain. Thus, most brain-targeted therapeutics are not effective. However, several brain delivery strategies have been developed to facilitate the permeation of nanotherapeutics through the BBB. Nanotherapeutics with sizes below 100 nm have unique multifunctional capacity to enhance the non-invasive delivery of drugs across the BBB into the brain, protect the therapeutic agent by encapsulating them into its core, control the drug release rate, target them to the site of therapeutic site of action without degradation, modify the drug’s PK and enhance their bioavailability [233]. As discussed in Section 5, having so many therapeutic targets on α-Syn makes it difficult to develop effective all-in-one therapeutic interventions. Furthermore, high expression levels of α-Syn is a popular intervention target in the treatment of PD. Antisense oligonucleotides (ASOs) can reduce α-Syn expression levels by silencing. However, delivering it effectively and safely into the neurons is a huge challenge because it binds to several targets and is degraded quite rapidly by proteases. Yang et al. developed an ASO4-loaded exosome nanoparticle delivery system (Exo-ASO4), which was injected intracerebroventricularly into the brains of α-Syn A53T transgenic PD mice model. Exo-ASO4 delivered ASO4 efficiently with low toxicity, significantly reduced the expression level of α-Syn, and halted the neurodegeneration in the mice [234]. However, it was an invasive procedure. In a similar study, Igartúa et al. utilized curcumin-loaded polyamidoamine (PAMAM) dendrimer nanoparticles, a polymer therapeutic to prevent aggregation of α-Syn in vitro. PAMAM was reported to enhance the solubility and stability of curcumin by internalizing it into the PAMAM’s core. Curcumin binds α-Syn preformed aggregates, which mitigates α-Syn toxicity; however, it is poorly soluble in water. PAMAM enhances its solubility and stability and controls its particle sizes to the nanometer range. In addition, it has intrinsic therapeutic effects, including anti-protein aggregation inhibiting β-amyloid aggregation. Thus, it might facilitate a synergistic anti-aggregation effect against α-Syn. It also has antibacterial, antiviral, and anti-inflammatory properties [235]. It is envisioned that rational formulation of nanoparticle-based drug delivery systems can be designed to cross BBB, target and sequester preformed aberrant α-Syn conformers, and prevent fibril formation. Examples of nanoparticle-based delivery systems that have been used in α-Syn related neuropathology are listed in Table 2 [234,236,237,238,239,240,241,242,243,244,245], and graphical representations of various nanoparticle-based brain delivery systems are presented in Figure 5.

## 6. Conclusions and Future Perspectives

The critical role of α-Syn in PD pathogenesis is well-established in the literature, and several research efforts supporting the therapeutic strategies to combat α-Syn toxicity with promising preclinical studies and early clinical trial results have been published. However, several therapeutic interventions in the development pipeline have failed to meet the specified clinical endpoints. Thus, the development of disease-modified immunotherapeutic agents for the prevention and treatment of PD remains an unmet clinical and scientific need. The complex, heterogeneous, and intrinsically disordered nature of α-Syn monomer predisposes it to spontaneous complex interactions with other proteins, lipids, and other molecules, leading to misfolding, mutation, and formation of inclusions and aggregates, which are hallmarks of PD. Thus, the endogenous α-Syn monomer, which is neuroprotective, switches to complex aggregates of several transient intermediate and stable conformers (oligomers, fibrils, and polymorphs), which penetrate the neurons, causing neuronal damage, membrane leakage, neuronal lysis, neuroinflammation, and neurodegeneration in PD pathogenesis. Presently, there is no therapeutic or immunotherapeutic agent that targets any of the transient neurotoxic intermediate conformers because they are subtle and difficult to detect and characterize. Therefore, elucidating the mechanism of aggregation and the characteristics of prime biomarkers of α-Syn conformer in vivo, and monitoring their progression over time in a biomarker-controlled clinical trial could expand the understanding of the nonintuitive behavior of various α-Syn conformers in PD pathogenesis which is critical to a successful development, clinical trials, and regulatory approval of novel neuro-immunotherapeutics. In conclusion, α-Syn monomers exhibit nonintuitive opposing effects in the brain, promoting neurogenesis at physiologic concentrations and neurotoxicity when switched to α-Syn conformer fibrils. Therefore, we posit that a multipronged approach of targeting the toxic α-Syn conformers while stabilizing the monomeric α-Syn within its therapeutic window and designing smart nanoparticle-based brain delivery systems would expand immunotherapeutic strategies for brain delivery. This approach would also provide a robust platform for identifying effective immunotherapeutic targets for the diagnosis, prevention, treatment, and disease progression monitoring strategies for PD, as well as enhancing the success rate of current and future clinical trials.

## Figures and Tables

**Table 1:** Immunotherapeutic agents currently in clinical trial for Parkinson’s disease.

Therapeutic Class	Agent/Compound	Target/Mechanism of Action	Clinical Trial Stage	Findings/Effect on α-Syn	Status	Clinical Trials Govt. ID (NTC #: National Clinical Trials Number)	Start Date	End Date	Sponsor/Collaborator	References
Active	Affitope-PD01A	Mimic α-Syn C-terminus; stimulate B cell antibody response; bypass T cell mobilization.	1	Decreased α-Syn oligomer in CSF; sustained IgG antibody response.	Completed, safe, and tolerable.	NCT02618941	February 2012	February 2017	AFFiRiS AG	Volc et al., 2020 [90]
	Affitope-PD03A	Activate T-helper cells; targets aggregated α-Syn	1	Decreased α-Syn oligomer in the cortex; rise in blood and CSF α-Syn antibodies	Completed, safe, and tolerable.	NCT02267434	December 2014	August 2016	AFFiRiS AG	Poewe et al., 2021 [231]
	UB-312	Mimic α-Syn C-terminus; activate T-helper cells	1/2	Prevent aberrant α-Syn spread; promote aggregate clearance	Completed, safe, and tolerable.	NCT04075318	August 2019	March 2023	Vaxxinity	Yu et al., 2022 [232]
Passive	Prasinezumab	α-Syn C-terminus interferes with truncation; promotes lysosomal clearance.	2/2b (Intervention)	Decreased concentration of free α-Syn down to 4%; selectively binds α-Syn aggregates;	Ongoing (missed the primary efficacy clinical endpoint on MDS-UPDRS global score)	NCT03100149	June 2017	September 2026 (primary end date was November 2019)	Hoffmann-La Roche	Pagano et al., 2022 [158]
	Cinpanemab (BIIB054)	Targets N-terminus of α-Syn aggregates	2	Binds free α-Syn at high concentrations; selectively binds α-Syn aggregates	Trial terminated in February 2021 because the primary and secondary endpoints were not met.	NCT03318523	January 2018	N/A: Not Applicable.	Biogen	Lang et al., 2022 [159]

**Table 2:** Nanoparticle-based immunotherapeutic brain delivery systems for Parkinson’s disease.

Class of Nanomaterial	Delivery System	Therapeutic Agent	Mechanism of Drug Delivery	Limitation	References
Lipid-based nanoparticles	Liposomes	N-3,4-Bis(pivaloyloxy)-dopamine	Stimulation of the striatum nigra; crosses the BBB; extended drug release	Not reported	Tacconelli et al., 2018 [236]
	Self-assembling nanomicellar system (SANS)	L-DOPA	Prolonged drug release; suitable for topical use	Lack of targeting ability	Sintov et al., 2017 [237]
	Polymeric micelles	None	Efficient BBB penetration; biocompatible	Long metabolic time	Yang et al., 2019 [238]
	Micelles	Baicalein	Sustained drug release; high payload; crosses BBB	Risk of mitochondrial damage	Chen et al., 2017 [239]
	PAMAM dendrimers	Carbamazepine	Extended drug circulation time; easily dissolved DDS, low toxicity	Acid- and alkaline-sensitive	Igartúa et al., 2018 [240]
	Exosomes	Dopamine; Antisense oligonucleotide	Crosses the BBB; low toxicity; promotes neurogenesis (neuroprotection); increases drug solubility; decreases neuroinflammation; reduces α-Syn expression; improves locomotor function	Unintended immune reactions by some exosomes; high cost	Qu et al., 2018 [241]; Yang et al., 2021 [234]
Polymeric nanomaterials	PLGA nanoparticles	Dopamine	Slow release and absorption; reduce ROS; low toxicity; extended circulation time;	Inflammatory response	Pahuja et al., 2015 [242]
Inorganic nanomaterials	Gold nanospheres	Xanthoceraside	High drug loading capacity; easily dissolved DDS	Lack of targeting ability	Meng et al., 2016 [243]
	Single-wall carbon nanotubes	Levodopa	High sensitivity; significant stability; minimum damage; real-time monitoring	Not reported	Ji et al., 2019 [244]
	SWCNT-PEGs-If	L-1,6-hydroxydopamine	Striatal targeting; excellent BBB crossing; high drug loading capacity; prolonged drug release; low toxicity; high biocompatibility	Inflammatory response	Guo et al., 2017 [245]

## Data Availability

Not applicable.
